# Measuring expression heterogeneity of single-cell cytoskeletal protein complexes

**DOI:** 10.1038/s41467-021-25212-3

**Published:** 2021-08-17

**Authors:** Julea Vlassakis, Louise L. Hansen, Ryo Higuchi-Sanabria, Yun Zhou, C. Kimberly Tsui, Andrew Dillin, Haiyan Huang, Amy E. Herr

**Affiliations:** 1 Department of Bioengineering, University of California Berkeley, Berkeley, CA USA; 2 Department of Molecular and Cell Biology, University of California Berkeley, Berkeley, CA USA; 3 Division of Biostatistics, University of California Berkeley, Berkeley, CA USA; 4 Howard Hughes Medical Institute, University of California Berkeley, Berkeley, CA USA; 5 Department of Statistics, University of California Berkeley, Berkeley, CA USA; 6 Center for Computational Biology, University of California Berkeley, Berkeley, CA USA

**Keywords:** Cytoskeletal proteins, Biochemical assays, Lab-on-a-chip, Assay systems

## Abstract

Multimeric cytoskeletal protein complexes orchestrate normal cellular function. However, protein-complex distributions in stressed, heterogeneous cell populations remain unknown. Cell staining and proximity-based methods have limited selectivity and/or sensitivity for endogenous multimeric protein-complex quantification from single cells. We introduce micro-arrayed, differential detergent fractionation to simultaneously detect protein complexes in hundreds of individual cells. Fractionation occurs by 60 s size-exclusion electrophoresis with protein complex-stabilizing buffer that minimizes depolymerization. Proteins are measured with a ~5-hour immunoassay. Co-detection of cytoskeletal protein complexes in U2OS cells treated with filamentous actin (F-actin) destabilizing Latrunculin A detects a unique subpopulation (~2%) exhibiting downregulated F-actin, but upregulated microtubules. Thus, some cells may upregulate other cytoskeletal complexes to counteract the stress of Latrunculin A treatment. We also sought to understand the effect of non-chemical stress on cellular heterogeneity of F-actin. We find heat shock may dysregulate filamentous and globular actin correlation. In this work, our assay overcomes selectivity limitations to biochemically quantify single-cell protein complexes perturbed with diverse stimuli.

## Abstract (web summary)

Existing methods for multimeric protein complex quantification in single cells suffer from limited selectivity and sensitivity. Here the authors report Single-cell protein Interaction Fractionation Through Electrophoresis and immunoassay Readout (SIFTER) and use this to probe the effects of cellular stress.

## Introduction

Over 80,000 protein complexes comprised of interacting proteins regulate processes from proteostasis to transcription^1^. A critical set of multimeric protein complexes form the cell cytoskeleton: actin filaments, microtubules, and intermediate filaments. For example, actin dynamically polymerizes and depolymerizes^2,3^ between monomeric G-actin (~42 kDa) and filamentous F-actin^4^ to determine cell morphology, motility, and proliferation^5^. F-actin is considered the functional actin species in the cytoskeleton. Thus, the F-actin ratio (or F-actin abundance divided by total actin) is a metric for cytoskeletal integrity. F-actin levels can be increased in metastatic cancer cells^5^, thus underpinning the design of oncology drugs that disrupt F-actin filaments^6^. In addition, microtubule-stabilizing chemotherapeutics (e.g., taxanes) are widely used in the treatment of numerous cancers^7^ (e.g., breast, lung, and prostate). However, the development of taxane-resistant cell subpopulations^8^ requires further advances to screen drugs that target the cytoskeleton. Quantifying the distribution of cytoskeletal protein complexes in single cells would inform drug development and help elucidate stress-induced cancer transformations.

To understand cytoskeletal protein-complex expression heterogeneity, no existing method combines the needed detection sensitivity, throughput, and selectivity for multimeric protein complexes in single cells. Single-cell, bottom-up mass spectrometry has been demonstrated^9,10^, with identification of up to 1000 protein groups from individual cells with the nanoPOTS system^11^. Bottom-up mass spectrometry digests proteins and cannot determine protein-complex stoichiometry like top-down mass spectrometry of intact proteins. However, lossy sample fractionation in top-down mass spectrometry limits the identification of protein complexes from low-cell number samples^12^. Indeed, with nanoPOTS integrated with top-down mass spectrometry, only ~170 of over a million possible proteoforms were detectable from ~70 pooled HeLa cells^13^. Thus, while highly multiplexed, top-down mass spectrometry currently lacks single-cell resolution for protein complexes. Targeted approaches such as proximity ligation assay and FRET achieve single-cell sensitivity and can assess cellular heterogeneity with flow cytometry readout (10,000 or more cells^14^). Proximity ligation assay and FRET rely on adjacent oligo-bound antibodies or fluorescent probes to infer that two proteins are interacting^15,16^. As proximity-based techniques are designed to measure 1:1 interaction, the most commonly used probes (whether fluorophores for FRET, or antibody-oligonucleotide conjugates for proximity ligation assay) do not distinguish multi-component or multimeric complexes from 1:1 complexes. Further, with flow cytometry, it is challenging to discern protein complexes from monomeric proteins without a selective probe. Finally, actin-specific detection methods are numerous, but suffer from limitations impacting sensitivity, selectivity, and applicability to other cytoskeletal protein complexes. Visualization of the actin cytoskeleton relies on fluorescently tagged actin (e.g., GFP-actin fusion or split GFP-actin fusion^17^), GFP-fused actin-binding proteins or peptides (e.g., Lifeact, F-tractin, and Utrophin), nanobodies^18^, or chemicals that directly bind actin (e.g., phalloidin). Such molecules may alter cytoskeletal dynamics both in vitro and in vivo^19–21^. Phalloidin competes with or is dissociated by, endogenous actin-binding proteins^22,23^ and actin-targeting drugs, such as Jasplakinolide^24^. Bulk ultracentrifugation overcomes these limitations while sacrificing single-cell resolution. In bulk ultracentrifugation, mild lysis in F-actin stabilization buffer solubilizes G-actin and preserves F-actin. The supernatant (G-actin) and pellet (F-actin) fractions are subsequently quantified by western blot or DNase inhibition assay^25^. However, bulk ultracentrifugation typically requires ~10^7^ cells, masking underlying cell-to-cell variation^25^.

In this work, we address gaps in multimeric protein-complex quantification by introducing Single-cell protein Interaction Fractionation Through Electrophoresis and immunoassay Readout, or SIFTER. With sequential differential detergent fractionation and bi-directional, single-cell polyacrylamide gel electrophoresis (originally developed for nuclear versus cytoplasmic protein separation^26^), we electrophoretically separate monomers from protein complexes. Single cells are gravity-settled in microwells formed in polyacrylamide, where the microwell aspect ratio is selected to maximize single-cell microwell occupancy^27^. Here, each cell is lysed in situ in a buffer designed to maintain protein complexes. Under an applied electric field, the gel size-excludes protein complexes larger than ~740 kDa in the microwell. Small monomeric proteins electromigrate into the gel in two steps: first from the monomeric fraction, and second from the intentionally depolymerized protein complex fraction after a buffer exchange. SIFTER fractionates protein complexes in <1 min, or 40× faster than the ultracentrifugation assay. The thin SIFTER gel (0.5 mm thick) minimizes resistive heating that could prematurely depolymerize or dissociate protein complexes. Owing to the arrayed format and open microfluidic design, hundreds of fractionation separations are performed simultaneously. After fractionation and bi-directional electrophoresis, both the depolymerized protein complex (e.g., F-actin, microtubule, and/or intermediate filament) and monomer (e.g., G-actin, β-tubulin, and/or vimentin) states are blotted (immobilized) in distinct gel regions abutting each microwell. Protein complex and monomer states are quantified by in-gel immunoprobing, allowing target multiplexing^27^. We applied SIFTER to four basic questions. First, do two well-studied actin-targeting drugs (Latrunculin A and Jasplakinolide) induce variation in F-actin complex-levels in single cells compared to controls? Second, as a corollary, does Latrunculin A yield cellular phenotypes distinct from controls with different organizations of other cytoskeletal protein complexes, such as microtubules and intermediate filaments? Third, what is the distribution of the F-actin ratio across a population of single cells? Fourth, how does heat shock, another cellular stress, shift the F-actin ratio distribution and coordination between F- and G-actin at the single-cell level? We show SIFTER is a versatile method for understanding cellular heterogeneity—at single-cell resolution—in protein-complex levels in response to perturbation.

## Results

### SIFTER design principles and characterization

**Fig. 1:**
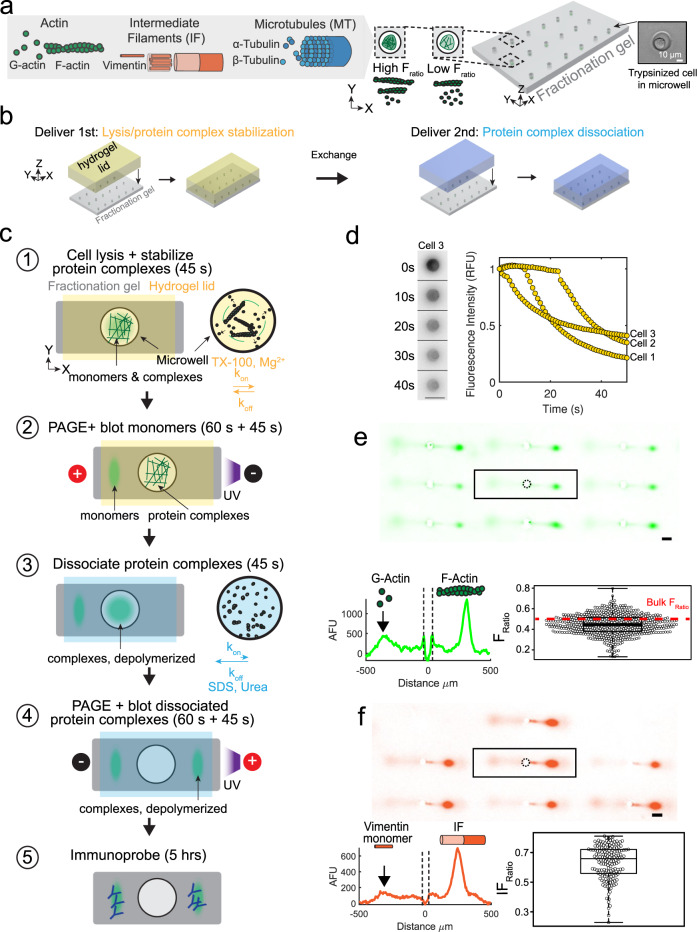
SIFTER detects cytoskeletal complexes from hundreds of single cells by on-chip integration of single-cell differential detergent fractionation and immunoblotting. **a** Schematic of three key cytoskeletal protein complexes: filamentous actin (F-actin; four to hundreds of globular G-actin, 42 kDa each), microtubules (MT; assembled from α- and β-tubulin heterodimers), and intermediate filaments (IF; comprising vimentin monomers). Trypsinized cells contain the three cytoskeletal protein complexes and are heterogeneous with low to high F-actin ratios (*F*_ratio_). Brightfield image shows a cell gravity settled into an individual microwell of a polyacrylamide fractionation gel. **b** Side-view schematic of hydrogel lid delivery of assay-stage optimized buffers to microwells in the fractionation gel. **c** The SIFTER assay comprises: (1) hydrogel lid delivery of protein complex-stabilizing lysis buffer to the array; (2) polyacrylamide gel electrophoresis (PAGE) and UV-immobilization of monomers (e.g., G-actin) in the gel; (3) hydrogel lid delivery of protein-complex dissociation buffer; (4) PAGE of dissociated protein complexes (e.g., F-actin, depolymerized) in the opposite direction of monomers and UV immobilization; and (5) in-gel antibody immunoprobing. **d** Cell lysis monitoring: false-color fluorescence micrograph montage and quantification of single MDA GFP-actin cells in microwells (cells 1−3) upon lysis with F-actin stabilization buffer (lyses cell but retains F-actin). The scale bar is 100 μm. Total fluorescence in the microwell normalized to initial in-microwell fluorescence as a function of lysis time for *n* = 3 cells (yellow circles). **e** Immunoassay results: representative false-color micrograph of a subset of the SIFTER array and intensity profile of GFP F-actin and GFP G-actin from single MDA-MB-231 GFP-actin cells (scale bar is 100 μm). Microwell annotated with a dashed line. Boxplot with beeswarm (white circles) shows the *F*_ratio_ calculated from F- and G-actin peak area-under-the-curve from *n* = 578 single-cell protein complex separations from *N* = 3 SIFTER devices. Boxplot box edges are at 25th and 75th percentile, the middle line is the median, and whiskers extend to the minimum and maximum values of the data set. The red dashed line shows the *F*_ratio_ from a bulk assay^38^. **f** Representative false-color micrograph of a subset of the SIFTER array and intensity profile of vimentin from single MDA-MB-231 GFP-actin cells (scale bar is 100 μm). Boxplot with beeswarm (white circles) shows *IF*_ratio_ = IF*/(*IF *+* *VIM*_monomer_*)*, from *n* = 168 cells from *N* = 4 SIFTER devices. Boxplot box edges are at 25th and 75th percentile, the middle line is the median, and whiskers extend to the minimum and maximum values of the data set. Source Data are available as a source data file.

To selectively detect cytoskeletal protein complexes from single cells, we integrate differential detergent fractionation, electrophoretic separation, and immunoassay steps into a single microdevice. An important set of dynamic protein complexes comprise the cytoskeleton, including F-actin filaments, microtubules (MT), and intermediate filaments (IF; Fig. 1a). Two design considerations are central to our measurement of dynamic protein complexes: (1) discerning the protein complexes from monomers, and (2) maintaining protein complexes during fractionation. For the first design consideration, we focus on the F-actin filament, which is the smallest and most dynamic of the three cytoskeletal protein complexes. Each filament can be composed of up to hundreds of globular G-actin monomers (*k*_off_ ~ 0.2–1.0 s^−1^ in vivo^28^). F-actin averages ~2.7 MDa (versus MT at ~178 MDa with 1 μm average MT length^29^ and 1625 tubulin heterodimers per μm of MT^30^, and IF at ~30 MDa for typical μm-scale IF^31^ at >30 kDa per nm of filament^32^). F-actin polymerization proceeds rapidly once four G-actin monomers are incorporated in a filament. Steady-state polymerization (*k*_on_ ~0.1−5 μM^−1^ s^−1^)^28^ yields a distribution of filament masses^33^. While the F-actin mass distribution below ~2700 kDa is unknown in vivo, F-actin is highly enmeshed. Thus, discerning F- (>160 kDa) vs. G-actin (42 kDa) requires coarse size cutoff (~hundreds of kDa), which should also fractionate MT and IF. On the second design consideration, rapid F-actin depolymerization occurs below the critical concentration of total actin (~0.2–2.0 μM in vivo). To maintain local concentrations of actin above the critical concentration demands < ~10× dilution during the assay, as cellular total actin is ~10−100 μM. Thus, the SIFTER fractionation gel contains microwells with ~10^8^× smaller reaction volume versus bulk ultracentrifugation to minimize dilution. The microwells accommodate gravity-sedimented single cells^27^ within the fractionation gel (Fig. 1a). The open SIFTER device is suited to the rapid serial introduction of buffers via interchangeable hydrogel lids to first lyse cells and stabilize protein complexes during fractionation, and then depolymerize or dissociate protein complexes to spatially separate monomers from protein complexes (Fig. 1b, c).

To report both the state (protein complex vs. monomer) and the amount of specific protein complexes per cell, SIFTER comprises five assay steps (Fig. 1c). First, single trypsinized cells are gravity-settled in the microwell array (from a cell suspension^27^) and lysed in an F-actin stabilization buffer delivered by the hydrogel lid, creating a lysate containing the monomers and complexes. Second, protein complexes are fractionated from the smaller monomers by polyacrylamide gel electrophoresis (PAGE, 60 s), during which large protein complexes are size-excluded from the gel and retained in each microwell. Monomers electrophorese into the gel and are immobilized (blotted) using a UV-induced covalent reaction to benzophenone methacrylamide integrated into the gel polymer network^27^. Covalent immobilization to the gel prevents monomer diffusion that would broaden the protein peak and result in protein loss out of the gel. Third, to depolymerize the complexes retained in each microwell, a protein-complex depolymerization buffer is introduced by another hydrogel lid. Fourth, we electrophorese the now depolymerized complexes into a region of the gel separate from the immobilized monomers, where depolymerized complexes are in turn immobilized. Fifth, in-gel immunoprobing (~5 h) detects the immobilized populations of monomer and monomer depolymerized from the complexes (Fig. 1e, f). We use a fluorescently labeled antibody probe against the protein (i.e., anti-actin antibody probe to detect F- and G-actin, and an anti-vimentin antibody probe to detect vimentin monomers and intermediate filaments).

To maintain intact protein complexes in each microwell during PAGE fractionation, the F-actin stabilization buffer slows the natural depolymerization kinetics. The non-ionic detergent Triton X-100 at ~1% v/v lyses the cell and minimally alters in vitro polymerization rates of actin^25,34,35^. The addition of 2 mM MgCl_2_ stabilizes F-actin complexes^25^, as Mg^2+^ binds G-actin to lower depolymerization rates^33^. Consequently, only ~2% of total F-actin depolymerizes per minute in mammalian cells lysed in stabilization buffer^25^, compatible with our goal to fractionate in ~1 min. Cell lysis depends on the diffusion of Triton X-100 micelles, which require ~10 s to reach the bottom of the microwells^36^. Imaging release of monomeric G-actin fused to fluorescent GFP from GFP-actin expressing breast cancer cells (MDA-MB-231 GFP-actin) within a microwell confirms a 45 s lysis yields only ~2.5−4× dilution of total actin to remain above the actin critical concentration (Fig. 1d). Important to minimizing F-actin-complex depolymerization during the assay, SIFTER completes cell lysis and fractionation in <5 min, or ~40× faster than bulk ultracentrifugation.

### Validation and benchmarking SIFTER

We first validated SIFTER by fractionating and quantifying the G-actin monomer vs. F-actin complexes in single MDA-MB-231 GFP-actin cells. We selected GFP-actin expressing cells to utilize fluorescence imaging to optimize cell lysis (Fig. 1d) and PAGE conditions. Immunoprobing for GFP yields distinct Gaussian protein peaks corresponding to GFP G-actin (G) on the left and GFP F-actin (F) to the right of each microwell (Fig. 1e). The area-under-the-curve of F-actin and G-actin peaks corresponds to the F-actin (F) and G-actin (G) protein fraction abundances, respectively. By design, the target peak is identified using a combination of reactivity with immunoprobe and migration distance (size). For immunoblots where dispersed signal between the target actin peak and microwell is both detectable and resolvable, the off-target signal is excluded from quantification. Immunoblots with non-Gaussian target signal are omitted from data analysis. We attribute the dispersed signal to either (or both): (i) cross-reactivity of the fluorescent antibody probes with smaller proteins and cellular material or (ii) injection dispersion arising from likely incomplete protein solubilization^37^ (including dissociation of the filamentous actin we study here). Full solubilization of the F-actin filament may not be complete for all cells in the short 45 s lysis and solubilization period, a duration that is dictated by diffusive losses of protein out of the microwell prior to electrophoresis^26^. We calculate the F-actin ratio:

1 $${F}_{{{{{{\mathrm{ratio}}}}}}}=F/(F+G)$$

for each cell. The MDA-MB-231 GFP-actin fusion cell average *F*_ratio_ = 0.45 ± 0.10 (standard deviation; *n* = 578 cells, from *N* = 3 SIFTER devices), in reasonable agreement with *F*_ratio_ ~0.5 for MDA-MB-231 from bulk ultracentrifugation^38^. With SIFTER, the *F*_ratio_ coefficient of variation is 22%, revealing single-cell variation obscured in the bulk assay. *F*_ratio_ variation measured by SIFTER includes cellular variation, such as the inverse correlation between the *F*_ratio_ and cell volume. For example, cells grown in microniches that controllably decrease cell volume by half undergo a similar magnitude increase in F-actin and decrease in G-actin (which should correspond to ~2× increase in *F*_ratio_)^39^.

Further, the F-actin stabilization buffer also maintains IF complexes (Fig. 1f, Supplementary Note 1). As such, we define and quantify an IF ratio*:*

2 $${{{{{\mathrm{{\it{IF}}}}}}}}_{{{{{{\mathrm{ratio}}}}}}}={{{{{\mathrm{IF}}}}}}/({{{{{\mathrm{IF}}}}}}+{{{{{\mathrm{{\it{VI{M}}}}}}}}}_{{{{{{\mathrm{monomer}}}}}}})$$

from the area-under-the-curve of the peaks, where *VIM*_monomer_ is the amount of native vimentin monomer and IF is the amount of depolymerized intermediate filament in arbitrary fluorescence units. The IF_ratio_ indicates the fraction of vimentin actively giving structure to the cell, the primary function of IF. We find MDA-MB-231 GFP-actin cells have an average *IF*_ratio_ = 0.63 ± 0.11 (error is the standard deviation; *n* = 168 cells, from *N* = 4 SIFTER devices measured on the same day). The significance of determining metrics such as *F*_ratio_ and *IF*_ratio_ with the single-cell resolution is to detect small sub-populations of cells with distinctive filament and monomer distributions, especially the phenotypes that arise in response to stresses. Observed cell-to-cell variation in *F*_ratio_ and *IF*_ratio_ raises the intriguing question of whether cells compensate levels of one cytoskeletal protein complex for another. We investigate differential stress responses and compensation of cytoskeletal protein complexes later in this work.

**Fig. 2:**
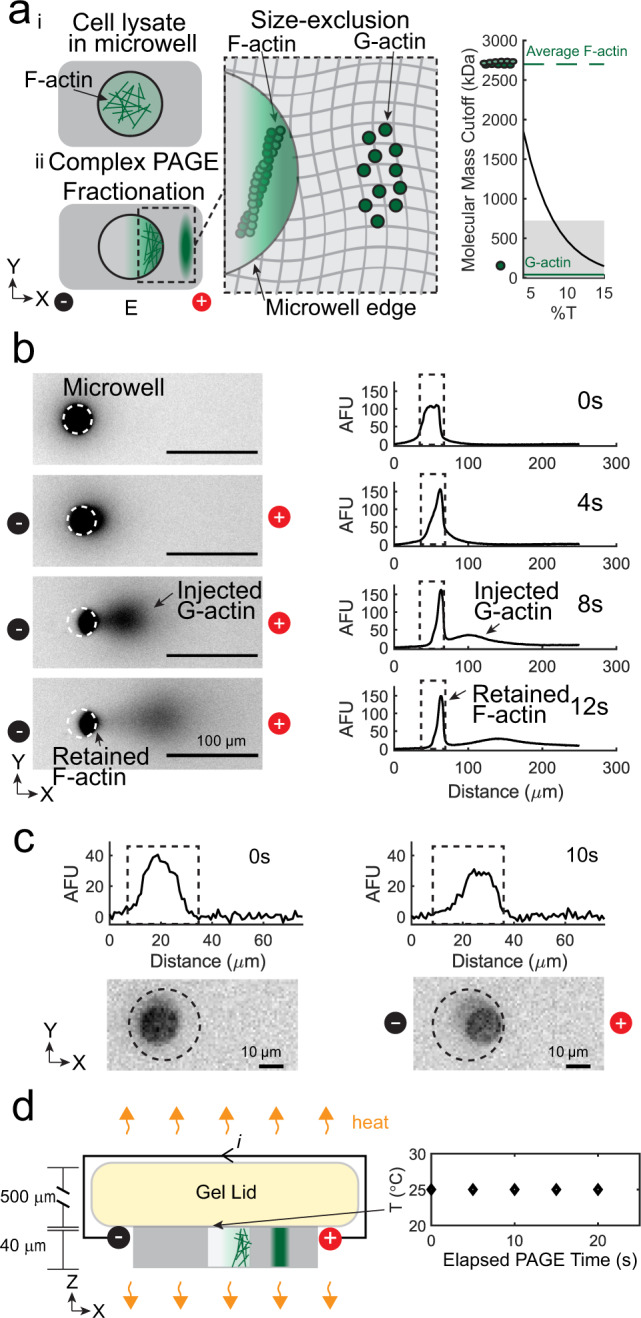
Size-based fractionation and efficient heat dissipation at the micro-scale provides molecular specificity to fractionate F-actin complexes from single cells. **a** Left: schematic of fractionation using polyacrylamide gel electrophoresis (PAGE) to separate F-actin complexes from G-actin monomers. Right: estimated molecular mass cutoff as a function of gel density (%T). The shaded region is the molecular mass range of 99.9% of non-interacting protein species comprising the mammalian proteome, with notations indicating G-actin (42 kDa, solid green line) and average F-actin (~2700 kDa, dashed green line) molecular masses. **b** False-color fluorescence micrographs and corresponding intensity profiles during electrophoresis (30 V cm^−1^) of MDA-MB-231 GFP-actin single-cell lysates in F-actin stabilization buffer; 76 ± 3% of the fluorescence remains in the microwell (*n* = 4, error is the standard deviation). Microwell is outlined with a dashed line in the micrograph and intensity profile. **c** Intensity profiles (top) and false-color fluorescence micrographs of single RFP-Lifeact U2OS cells in microwells (dashed outline; only F-actin is fluorescent) upon lysis in F-actin stabilization buffer. PAGE results in retention of F-actin complexes in the microwell (repeated for a total of *n* = 3 cells). **d** Left: schematic of heating in the fractionation gel (gray) and gel lid (yellow) upon applying a current, *i*. Right: plot of temperature as a function of elapsed PAGE time under the F-actin stabilization lysis buffer gel lid at 30 V cm^−1^ (*n* = 3; black diamonds). Source Data are available as a source data file.

To validate monomer vs. protein-complex detection specificity, we determined the gel composition needed to fractionate F-actin (the smallest of the three cytoskeletal protein complexes) and directly observed PAGE of fluorescently labeled actin from single-cell lysates. The molecular mass cutoff for the gel depends on the total acrylamide concentration (%T). Based on native PAGE^40,41^, the SIFTER cutoff for an 8%T gel is ~740 kDa (Supplementary Fig. S1 and Fig. 2a), or larger than 42 kDa G-actin, but smaller than an average ~2700 kDa F-actin. During PAGE of MDA-MB-231 GFP-actin cells (in which GFP is fused to both G- and F-actin), actin species indeed fractionate at the microwell edge (Fig. 2b). Within 45 s of PAGE, the G-actin Gaussian protein band completely injects a mean distance of 350 ± 16 μm into the polyacrylamide gel (with mean peak width of 66 ± 8 μm, *n* = 275; errors are standard deviations from one SIFTER device). We confirm the actin state of the species in the microwell by imaging PAGE of U2OS cells expressing RFP-Lifeact, a common marker for F-actin^19^. The microwell retains the F-actin complexes (Fig. 2c), with signal decrease attributable to diffusive losses^27^ of RFP-Lifeact-bound G-actin out of the microwell and photobleaching. We hypothesize two factors lead to no observed F-actin electromigration into the gel, including RFP-Lifeact bound dimers^42^. First, small oligomers are a minor fraction of F-actin due to substantial dissociation rates^43^. Second, highly crosslinked filaments^23^ remain enmeshed within the cytoskeleton even in lysed cells^44^. Further, we expect that free RFP-Lifeact would diffuse out of the microwell during cell lysis if present. Thus, we confirm that SIFTER fractionates F-actin complexes from single cells. Importantly, size exclusion may fractionate other protein complexes by adjusting the %T, as >99% of individual proteins of the mammalian proteome would not be size-excluded by a smaller pore-size 10%T gel^45^.

We further validate SIFTER maintains F-actin complexes during fractionation without PAGE-induced temperature rise that may depolymerize or dissociate protein complexes (e.g., as shown in vitro or in certain cell types above 45 °C^46–48^). An electrical current passing through conductive buffer produces heat (Joule heating) during PAGE, which can increase the temperature if not efficiently dissipated. The temperature difference, Δ*T*, between the surrounding medium and the conductor varies along the height axis, *z*, of the conductor:

3 $$\varDelta T={E}^{2}{\sigma }_{c}\left(\frac{{a}^{2}-{z}^{2}}{2k}\right)$$

where *E* is the electric field strength (V m^−1^), *σ*_*c*_ is the buffer conductivity (S m^−1^), 2*a* is the height and *k* is the thermal conductivity of the conductor (W m^−1^ C^−1^)^49^. Due to large temperature rises during electrophoresis in F-actin stabilization buffers containing MgCl_2_ (*σ*_*c*_ ~0.13 S m^−1^), *E* is limited to ~2−10 V cm^−1^ for 120−480 min in native slab gels^50^, or ~18 V cm^−1^ in capillaries^50^. In SIFTER, the anticipated Δ*T* at 30 V cm^−1^ is ~0.02 °C (2*a* ~ .54 mm) vs. ~6.18 °C increase in a slab gel (2*a* ~ 5 mm; Supplementary Fig. S2). Indeed, we measure constant room temperature using liquid crystal temperature sensors under the hydrogel lid during PAGE at 30 V cm^−1^ with SIFTER (Fig. 2d). Thus, we confirm SIFTER maintains endogenous protein complexes without Joule heating with ~100× faster fractionation than in a slab gel, 100−1000× higher sample throughput than a capillary (or comparable to automated capillary systems^51^), and without purifying, labeling, or crosslinking of complexes^52^.

We sought to validate SIFTER’s quantification of single-cell heterogeneity of F-actin complex levels as quantitative assessment is needed for screening drugs targeting metastatic cell subpopulations^53^. In conventional imaging of F-actin with phalloidin (conjugated to a fluorophore), two factors pose a challenge to quantifying F-actin complex heterogeneity. First, phalloidin competes with, or is dissociated from, F-actin by both actin-binding proteins (e.g., cofilin)^22,23^ and drugs (e.g., actin nucleating drug jasplakinolide, Jpk^24^, and the structurally similar MiuA^54^). The number of potential actin-targeting drugs that compete with phalloidin are unknown. Nevertheless, Jpk and MiuA highlight the fact that a decrease in phalloidin staining signal can be due to decreased F-actin expression, competitive binding, or a combination of the two. Second, optimal cell segmentation requires that cells are not in contact with one another^55^, which limits quantification from tissues and high-throughput analysis^55^. The latter may be overcome in the case of actin by conducting analysis by flow cytometry. While flow cytometry is compatible with proximity ligation assay for two proximal proteins, the lack of antibodies specific for protein interactions prevents multi-component protein complex measurement by flow cytometry^56^. Alternatively, SIFTER is free from competitive binding, cell segmentation challenges, and can discern and quantify protein complexes.

**Fig. 3:**
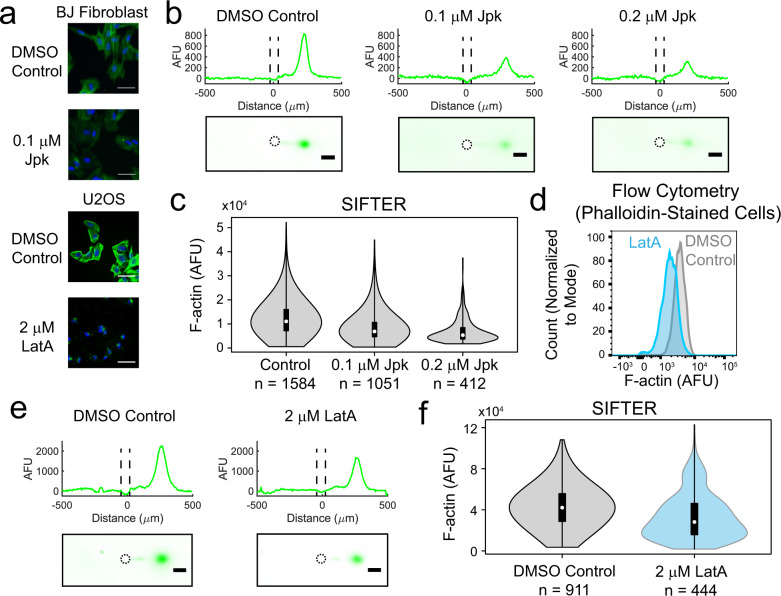
SIFTER quantifies cellular heterogeneity in F-actin complex levels, avoiding competitive binding or cell segmentation challenges encountered with phalloidin staining and capturing the cellular variation identified by flow cytometry. **a** False-color fluorescence micrographs of U2OS or BJ fibroblast cells fixed and stained with fluorescent phalloidin (F-actin, green) and Hoechst (nuclear stain, blue) after incubation with Latrunculin A (LatA, 60 min) or Jasplakinolide (Jpk, 120 min). The scale bar is 50 μm. The experiment was repeated a total of two times. **b** False-color fluorescence micrographs and representative intensity profiles from SIFTER on single BJ fibroblast cells treated with the indicated concentration of Jpk. The scale bar is 100 μm. Microwell is outlined with a dashed line in the intensity profile and in the micrograph. **c** Violin plot of F-actin levels quantified from three different SIFTER devices with the indicated total number of single cells. Median F-actin AFUs are 11053 for control, 6876 for 100 nM Jpk, and 5343 for 200 nM Jpk. Boxplot box edges are at 25th and 75th percentile, the middle point is the median, and whiskers extend to the minimum and maximum values of the data set. Kruskal−Wallis *p*-value < 0.0001 with Dunn−Sidak correction for multiple comparisons. **d** Histograms of F-actin fluorescence with cell count normalized to the mode from flow cytometry measurement of trypsinized and phalloidin-stained U2OS cells. Median F-actin AFUs are 3454 for control (*n* = 9203, gray) and 1858 for LatA (*n* = 5114, blue). Mann−Whitney U Test *p*-value < 0.0001. **e** False-color fluorescence micrographs and representative intensity profiles from performing SIFTER on single U2OS cells. The scale bar is 100 μm. Microwell is outlined with a dashed line in the intensity profile and in the micrograph. **f** Violin plot of F-actin levels quantified from four different SIFTER devices with the indicated total number of single cells. Boxplot box edges are at 25th and 75th percentile, the middle point is the median, and whiskers extend to the minimum and maximum values of the data set. Medians are 42105 for control (*n* = 911, gray) and 28144 (*n* = 444, blue) for LatA. Mann−Whitney U test *p*-value < 0.0001. Source Data are available as a source data file.

With SIFTER, we investigated two well-studied drugs—Jpk and Latrunculin A (LatA)—each having a unique mechanism of action on actin^57,58^ (Fig. 3). Understanding Jpk effects on F-actin complexes is confounded by competitive binding with phalloidin and differing observations in vivo versus in vitro^58^. Jpk binds at the interface of three actin subunits^59^ to lower the number of actin subunits needed for a stable multimer for filament elongation, causing disordered aggregates^58^. Still, F-actin complex levels increase in certain cell types with Jpk treatment in the 0.1−1.0 μM range as determined by bulk ultracentrifugation^60,61^. With phalloidin staining of Jpk-treated BJ fibroblasts, we qualitatively observe shorter filaments and small aggregates when dosing with 100 nM Jpk. While 100 nM Jpk is below the reported IC_50_ of 555 nM Jpk treatment (for 4 h in breast cancer cells)^62^, the concentration is high enough to induce decreased phalloidin fluorescence. When phalloidin-stained Jpk-treated cells display decreased fluorescence signal, as with the BJ fibroblasts here, it is difficult to discern if competition with phalloidin obscures interpretation (Fig. 3a). SIFTER yields a ~1.6× and 2.1× decrease in median F-actin relative to the control at the 100 and 200 nM Jpk concentrations, respectively (Kruskal−Wallis *p*-value < 0.0001 with Dunn−Sidak correction for multiple comparisons, Fig. 3b, c). The decrease in F-actin levels from Jpk treatment is not accompanied by increasing G-actin immunoprobe signal, as described in Supplementary Note 3. To assess heterogeneity in SIFTER F-actin complex levels across hundreds to thousands of individual cells, we calculate the coefficient of quartile variation (CQV). The CQV is a metric of variance accounting for skewed distributions^63^, such as gamma-distributed protein expression^64^:

4 $${{{{{\mathrm{CQV}}}}}}=\frac{{Q}_{3}-{Q}_{1}}{{Q}_{3}+{Q}_{1}}$$

where *Q*_3_ is the 75th percentile and *Q*_1_ is the 25th percentile F-actin level. We find CQV_DMSO control, BJ_ = 0.38, CQV_Jpk 100 nM, BJ_ = 0.39, and CQV_Jpk 200 nM, BJ_ = 0.39 (subscripts refer to the treatment and cell type). Similar CQV values with increasing drug concentration indicate the drug effect is relatively consistent across the cell population.

In contrast, LatA sequesters G-actin and reduces both F-actin complex levels and the *F*_ratio,_ as determined by phalloidin staining, DNAse I staining (of G-actin), and bulk methods^57,65,66^, but variation in cell response is unknown. After treatment with 2 μM LatA, we phalloidin-stained U2OS cells and observed decreased F-actin complex fluorescence (Fig. 3a) in agreement with previous findings^66^. As with Jpk, we utilized a high enough LatA drug concentration to induce visible changes in phalloidin fluorescence. To assess variation in cell response to LatA, we benchmarked the distribution of F-actin levels from LatA treatment in SIFTER versus flow cytometry of trypsinized, fixed, and phalloidin-stained U2OS cells. By flow cytometry, we find the median F-actin complex level of DMSO control cells is significantly higher than the LatA treatment median by 1.9× (Mann−Whitney *P*-value < 0.0001, Fig. 3d, *n* = 9203 control cells, and *n* = 5114 LatA-treated cells). With SIFTER, we observe the median F-actin complex level in DMSO control cells is significantly higher than the LatA treatment median by 1.5× (Mann−Whitney *P*-value < 0.0001, *n* = 911 control cells, and *n* = 444 LatA-treated cells, Fig. 3e, f). We further found that SIFTER measured a significantly lower log-fold change (i.e., smaller increase in DMSO control over LatA) than flow cytometry (Mann−Whitney *P*-value < 0.0001, Supplementary Fig. S3 and Supplementary Note 2). Thus, SIFTER does not measure as large a decrease in F-actin levels upon LatA treatment as flow cytometry of fixed and phalloidin-stained cells. One reason SIFTER may report smaller decreases in F-actin levels upon LatA treatment (while still maintaining statistical significance) is due to run-to-run variation observed across assay replicates (each replicate shown in Supplementary Fig. S4).

Unlike Jpk, LatA treatment corresponds with an increase in F-actin CQV as CQV_LatA, U2OS_ = 0.49 vs. CQV_DMSO control, U2OS_ = 0.32 by SIFTER (a 1.5× increase) and CQV_LatA, U2OS_ = 0.30 vs. CQV_DMSO control, U2OS_ = 0.23 by flow cytometry (a 1.3× increase). Previously, phalloidin staining revealed a single F-actin complex-phenotype from ~200 sparsely seeded cells treated with 250 nM LatA^67^. Here, the CQV increase upon LatA exposure suggests differential cell tolerance to LatA potentially due to the almost 10× higher LatA concentrations utilized here. Thus, SIFTER circumvents competitive binding or cell segmentation challenges to quantify variation in drug effects on F-actin complexes at the single-cell level. The high CQV_LatA, U2OS_ from SIFTER prompted us to further investigate cellular variation in response to LatA treatment. It is not currently possible to quantify the variation in the other cytoskeletal protein complexes, IF and MT with flow cytometry, as vimentin and tubulin antibodies would bind both the monomer and protein complexes in the cell. However, with SIFTER, co-detection of protein complexes within the same cell is possible, using antibodies raised against different species, or with a chemical stripping and re-probing approach developed previously^27^.

### Multiplexed SIFTER uncovers subpopulations of LatA-treated cells

**Fig. 4:**
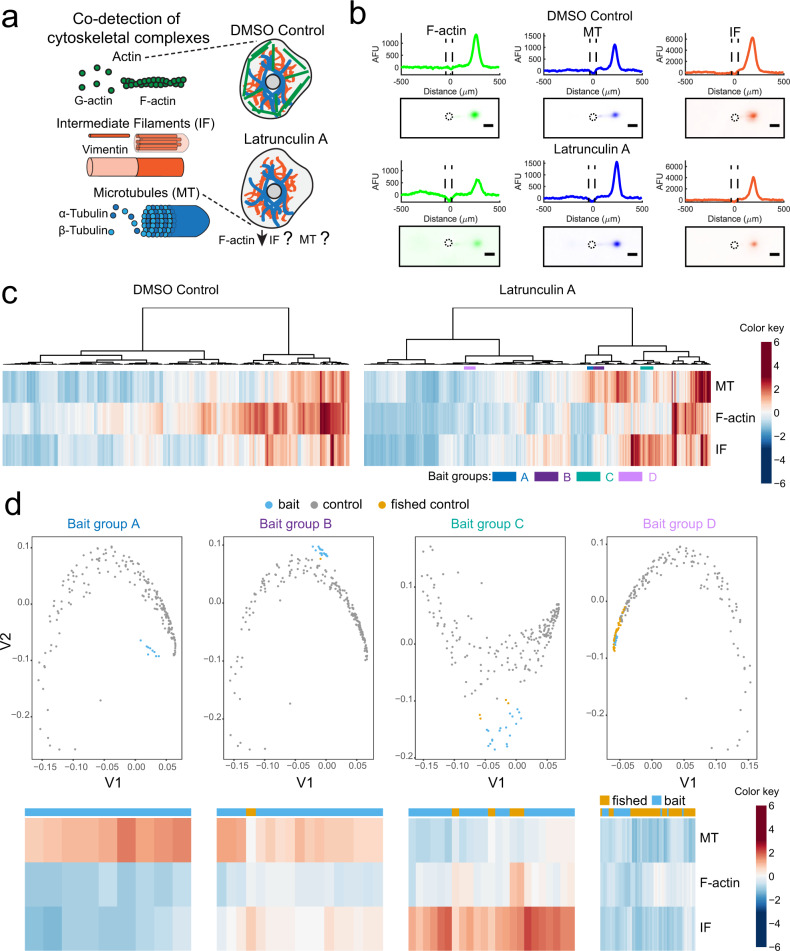
Multiplexed SIFTER detects subpopulations of cells with altered cytoskeletal protein complexes in response to F-actin destabilization. **a** Schematic of the cell cytoskeleton composed of F-actin, intermediate filaments (IF) and microtubules (MT), and the unknown effects of Latrunculin A (LatA) on IF and MT. **b** Representative false-color fluorescence micrographs and intensity profiles from SIFTER. F-actin (green), MT (blue), and IF (orange) are electrophoresed to the right of the microwell. Protein quantification is performed by peak area integration. The scale bar is 100 μm. Microwell is outlined by a dashed line in the micrographs and intensity profiles. **c** Heat maps with dendrograms from agglomerative hierarchical clustering with Euclidean distance metric and Ward linkage for U2OS cells incubated in DMSO (*n* = 201 cells, three SIFTER devices) or 2 μM LatA (*n* = 507 cells, four SIFTER devices). Distinct sub-lineages used as bait groups A-D for CellFishing are shown with colored bars (blue, purple, teal, and lavender, respectively). Heatmap is standardized by row (mean at 0, and color gradations at units of standard deviation). **d** Spectral clustering projections and heatmaps depicting LatA treatment bait group cells (blue), DMSO control cells (gray), and fished out DMSO control cells (yellow). Source Data are available as a source data file.

We asked two questions regarding LatA-induced cellular variation, recognizing that SIFTER could permit the measurement of all three major cytoskeletal protein complexes simultaneously. First, we sought to understand if LatA yields a differential expression of other cytoskeletal protein complexes. Second, we asked whether LatA induced unique cell subpopulations. The cytoskeletal protein complexes F-actin, microtubules (MT, of α- and β-tubulin subunits), and intermediate filaments (IF, of vimentin or keratin subunits) have both redundant and distinct functions in maintaining cytoskeletal integrity (Fig. 4a). Such redundancy^68^ yields increased IF to counteract F-actin destabilization of mesenchymal cells^69^ with only 1 h treatment with another Latrunculin, LatB. The counteracting increase in intermediate filament levels occurs because keratin intermediate filament-regulating genes become differentially expressed^69^. Yet, quantification of cytoskeletal changes remains a challenge in single cells by microscopy due to segmentation artifacts and low signal-to-noise ratio (SNR) from immunohistochemistry and phalloidin staining^70,71^.

To understand the concerted effects of 1 h LatA drug treatment on F-actin, MT, and IF, we performed same-cell, target-multiplexed SIFTER (Fig. 4b and Supplementary Fig. S5). We assess the expression relationships between the three protein complexes in the DMSO vehicle control cells (*n* = 201 single cells) by Spearman rank correlation, and obtain *ρ* = 0.70 for MT vs. F-actin, *ρ* = 0.72 for F-actin vs. IF, and *ρ* = 0.59 for MT vs. IF (Supplementary Fig. S6; *p* < 0.0001 for each correlation). The correlation values suggest the coordination of cytoskeletal protein-complex levels across a large proportion of cells. A follow-up agglomerative hierarchical clustering analysis reveals sets of cells with distinct patterns of protein-complex expression (e.g., groups A−D, Fig. 4c).

Next, to elucidate whether any of the potential subpopulations shown in Fig. 4c (e.g., groups A−D) were unique to the LatA-treatment, we adapted the GeneFishing method^72^ for CellFishing. Using a group of co-expressed cells as bait, we attempt to fish out other cells from a candidate pool that present a similar protein complex-expression pattern to that of the bait cells. We do this through a semi-supervised clustering approach, coupled with sub-sampling to ensure robust discoveries. Here, groups of LatA-treated cells from hierarchical clustering that appear as unique phenotypes each define a set of bait cells, and the DMSO control cells define the candidate pool. If a group of bait cells does not identify any cells with similar phenotypes in the DMSO control cells, we assume the phenotype is unique to the LatA-treated cell population. We found that bait group A does not fish out DMSO control cells, while groups B-D are examples of bait groups that do (Fig. 4d). Groups B (~3% of LatA cells), C (~4% of LatA cells), and D (~3% of LatA cells) all fish out DMSO control cells (~0.5, 2, and 20% of the DMSO control cells, respectively) and thus represent phenotypes not exclusive to LatA treatment. Group B is marked by elevated MT and to a lesser extent, elevated IF compared to the average protein complex expression levels of the LatA-treated cells. Group C is characterized by increased IF. Group D expresses low F-actin, MT, and IF, which was a phenotype observed in a substantial number of both control and LatA cells as displayed in the heat map. Group A (~2% of LatA-treated cells) is characterized by elevated MT in response to F-actin destabilization and is only found in the LatA treatment cells. If MT compensates for F-actin perturbation in subpopulations of cells, such cells may be better equipped to maintain cytoskeletal integrity in response to stress. LatA causes an increase in the percentage of serum-starved fibroblasts expressing mature microtubules (from 40 to 70% of cells after one hour at 0.1 μM)^73^, a shift between two cell populations. Here, hierarchical clustering of multiplexed SIFTER reveals distinct subpopulations with unique cytoskeletal composition stratified by the expression of all three complexes. For example, IF levels distinguish Groups A and B with similar MT and F-actin levels (Fig. 4c, d). Our results open up questions such as whether increases in MT in LatA-treated cells correspond with changes in transcriptional or translational rates, and subunit stability or MT organization, which warrant further investigation.

### Quantifying distributions of total actin and *F*_ratio_ across cells

To assess actin cellular heterogeneity, we asked: what are the statistical distributions of total actin and *F*_ratio_ across cells? In order to assess statistical distributions across SIFTER replicates, we needed to measure the cells at a fixed time after preparing the single-cell suspension, as detachment lowers the level of cytoskeletal protein complexes^74–76^. We conducted SIFTER replicates with constant cell handling times and measured the *F*_ratio_ from each device. The median *F*_ratio_ values from the three SIFTER replicates were 0.47, 0.42 and 0.46 (*n* = 275, *n* = 193, and *n* = 110 respectively, Kruskal−Wallis *p*-value = 0.0084; Supplementary Fig. S7), with a mean median of 0.45 and coefficient of variation of mean median of 5%. The interquartile ranges were 0.15, 0.13, and 0.08, which indicates the distributions overlap substantially with similar medians despite statistically significant run-to-run variation indicated by the *p*-value < 0.05. In each of three replicates displayed as quantile-quantile (QQ) plots, we found total actin largely follows a gamma distribution, as expected based on transcriptional bursting (Supplementary Fig. S8)^77^. One of the replicates deviates from the gamma distribution at the highest quantiles, indicating the tail behavior is less well-described by a gamma distribution. We find the *F*_ratio_ follows a normal distribution across cells by examining the QQ plots (Supplementary Fig. S8). The normal *F*_ratio_ distribution measured with SIFTER suggests actin-binding proteins stochastically regulate actin polymerization/depolymerization.

Characterizing *F*_ratio_ requires accurate quantification of the G-actin fraction to calculate the total actin (the denominator of the ratio, *F* + *G*). As with any immunoassay, immunoreagents must be screened for each specific application, as sample preparation determines whether the epitope is native, partially denatured, or fully denatured^78^ (Supplementary Table S1, Supplementary Note 3, and Supplementary Fig. S9).

### Heat shock induces cellular heterogeneity in actin distribution

**Fig. 5:**
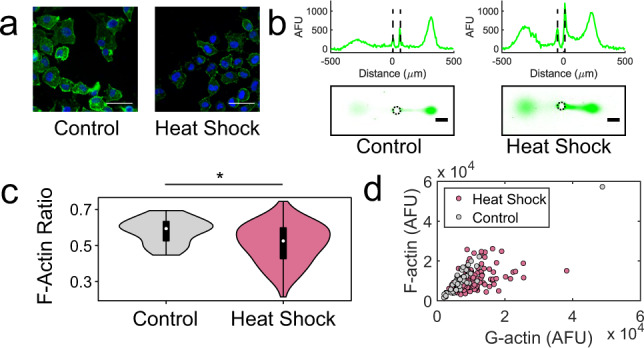
SIFTER quantifies actin distribution heterogeneity after heat-shock stress. **a** False-color fluorescence micrographs of adherent MDA-MB-231 GFP-actin cells with RFP-lentiviral transfection that were fixed and stained for F-actin (phalloidin, green) and the nucleus (Hoechst, blue) with heat shock (45 °C for 60 min) or 37 °C control. The scale bar is 50 μm. The experiment was repeated for a total of two times. **b** Representative false-color fluorescence micrographs and intensity profiles of GFP-actin PAGE fractionation from the specified single cells. The scale bar is 100 μm. Microwell is outlined by a dashed line in the intensity profiles and micrographs. **c** Violin plots of F-actin ratio, *F*/(*F* + *G*), from SIFTER with *n* = 49 for the control (one SIFTER device, gray) and *n* = 131 for the heat shock condition (two SIFTER devices, pink). Median *F*_ratio_ is 0.59 for control and 0.53 for heat shock. Boxplot box edges are at 25th and 75th percentile, the middle point is the median, and whiskers extend to the minimum and maximum values of the data set. Mann−Whitney (two-sided test) *p*-value is significant (*) at *p* < 0.0001. **d** Scatter plot of F- versus G-actin for control (gray circles) and heat shock (pink circles). Spearman *ρ* = 0.82 for control (*p* < 0.0001) and *ρ* = 0.47 for heat shock (*p* < 0.0001). Source Data are available as a source data file.

To assess how a non-chemical stress perturbs (1) the *F*_ratio_ distribution and (2) F- and G-actin coordination, we apply SIFTER to the study of heat shock. Cytoskeletal reorganization is a hallmark of disease states^5^, and protein-complex dysfunction is prominent in aging^79^ and during cellular stress^80,81^. Cell stress such as heat shock yields re-organization of F-actin in many, but not all cell types^82^. Indeed, with phalloidin staining, we observed a qualitative decrease in F-actin fluorescence of RFP-Lentiviral transformed MDA-MB-231 GFP-actin cells upon heat shock (Fig. 5a).

For more nuanced characterization of the *F*_ratio_ distribution not possible with phalloidin staining, SIFTER reports the median *F*_ratio_ in the heat-shocked cells was similar to control cells (0.53 vs. 0.59, respectively; Mann−Whitney *p*-value < 0.0001, Fig. 5b, c). However, the interquartile range of the *F*_ratio_ in heat-shocked cells is ~1.5× that of control cells (0.17 vs. 0.11). We quantified the skew of the distribution with the Pearson’s moment coefficient of skew:

5 $${\bar{\mu }}_{3}=\varepsilon \left[{\left(\frac{X-\mu }{\sigma }\right)}^{3}\right]$$

where *ε* is the expectation operator, *X* is the random variable (here, *F*_ratio_), *μ* is the distribution mean and *σ* is the standard deviation. We find ${\bar{\mu }}_{3}$ is −0.40 for the control data set, and −0.38 for the heat-shocked cells.

To understand if F- and G-actin levels remain coordinated upon heat shock, we quantified Spearman *ρ* (for F- and G-actin level correlation, Fig. 5d). The Spearman *ρ* decreased from 0.82 for the 49 control cells from one SIFTER device to 0.47 for 131 heat-shocked cells from two SIFTER devices. Across the two heat shock SIFTER devices, *ρ* = 0.73 for device 1 (*n* = 22, *p* = 0.0002) and *ρ* = 0.41 for device 2 (*n* = 109, *p* < 0.0001). We hypothesize heat shock SIFTER device 1 had too few cells that passed analysis quality control to capture the reduced correlation in F- and G-actin upon heat shock observed in device 2. We conclude that F-actin levels alone may not reveal actin cytoskeletal integrity: the Spearman *ρ* correlation of F- and G-actin also helps uncover differential stress response across the cell population.

## Discussion

SIFTER maintains multimeric cytoskeletal protein complexes during fractionation to reveal monomer versus protein-complex states in single cells. From perturbation of actin with well-characterized drugs, we find LatA, but not Jpk (at the concentrations tested), results in increased F-actin expression heterogeneity as characterized by increasing CQV. To investigate the heterogeneity of LatA-treated cells, we extended SIFTER to a multiplexed readout of the three major cytoskeletal protein complexes (F-actin, microtubules, and intermediate filaments) simultaneously in each cell. We identified previously unknown cell subpopulations, such as the cluster with decreased F-actin and potentially compensatory increases in microtubules (Group A) along with intermediate filaments (Group B) upon LatA treatment. Thus, observed heterogeneity in LatA F-actin response corresponds with a spectrum of cytoskeletal integrity in the clonal population of U2OS cells investigated. While some cells increase the expression of microtubules, intermediate filaments, or both, other cells in the population undergo a complete cytoskeletal collapse. In the clonal population of U2OS osteosarcoma cells investigated here, the origins of differential maintenance of the cytoskeleton are unknown. However, recent single-cell sequencing studies of U2OS cells identified coordinated expression of sets of genes across subsets of cells, including some genes that regulate the cytoskeleton^83^. Determining if MT- and IF-regulating genes are differentially expressed in subsets of U2OS cells and immunoprobing for the corresponding proteins along with F-actin, MT and IF in SIFTER could provide mechanistic insight. Partially coordinated regulation of the cytoskeleton raises two further questions: (1) what causes such differential gene expression to regulate the cytoskeleton in certain cells of a clonal population and (2) what is the functional implication of subsets of cells having a more resilient cytoskeleton? For the latter, we consider that the epithelial to mesenchymal transition of metastasis is marked by re-organization of the key cytoskeletal protein complexes^7^, and osteosarcoma is known for aggressive metastasis. Consequently, it is intriguing to consider whether cell subpopulations with compensating overexpression of microtubules and intermediate filaments (marked capability to re-organize the cytoskeleton) represent a more mesenchymal-like subtype.

Applying SIFTER to single-cell *F*_ratio_ assessment, we determined that the F-actin ratio is normally distributed across a cell population. This indicates the possibility that the F-actin ratio could be a metric for assessing whether a population of cells is at an equilibrium state in terms of actin distribution. To investigate a non-chemical stress, we evaluated the impact of heat shock on the F-actin ratio of cells. Though missed by phalloidin staining, SIFTER uncovers a potentially marked decrease in the correlation of F- and G-actin upon heat shock. Our results present the possibility that SIFTER presents a more nuanced assessment of actin cytoskeletal integrity than phalloidin staining.

Cellular stresses, be they chemical, heat shock, hypoxia, or oxidative stress, are critical features of cancer biology. Understanding which protein complexes are differentially expressed in drug-susceptible versus drug-resistant cells, or in subsets of cells that metastasize will be critical to advancing cancer therapies. Thus, SIFTER unlocks the capability to assess single-cell heterogeneity in the expression of multimeric protein complexes, with broad applications across biology, potentially including protein complexes unrelated to the cytoskeleton.

The SIFTER assay presently is conducted with a well-characterized F-actin stabilization buffer for cell lysis and maintenance of cytoskeletal protein complexes during electrophoresis. However, no single buffer is ideal for the stabilization of all protein complexes, prompting careful optimization of detergent, salt (ionic species and concentration), buffer, and pH for immunoprecipitation of specific sets of protein complexes^84^. We have not yet investigated alternative lysis buffers for SIFTER, such as certain immunoprecipitation buffers (e.g., containing 10−100 mM NaCl or KCl). Higher buffer salt concentrations than the F-actin stabilization buffer will increase buffer conductivity and we hypothesize could yield more extensive Joule heating that can dissociate protein complexes. Fabrication of thinner (<500 μm) hydrogel lids for efficient heat dissipation may be needed for PAGE in high salt buffers. Thus, further device or buffer optimization may be required to apply SIFTER to protein complexes beyond the cytoskeletal complexes investigated here.

The range of detectable and separable protein-complex sizes is set by a tradeoff between fractionation and immunoprobing. Denser gels compromise assay detection sensitivity because size-exclusion-based partitioning lowers the in-gel antibody probe concentration during the immunoassay^27^. Fractionation in decrosslinkable gel^85^ should allow isolation of up to hundreds of the known mammalian protein complexes with masses of ~295 kDa or greater in a 12%T gel (~7 or more protein subunits^86^, assuming each subunit has the average mammalian protein size of 375 amino acids^87^, or mass of ~40 kDa).

Another factor that determines which protein complexes are detectable with SIFTER is assay detection sensitivity. The cytoskeletal protein complexes investigated here are among the most abundant proteins in mammalian cells, often expressed at millions of copies^45^. Utilizing an in-gel immunoassay for readout, we have previously detected down to 27,000 copies of protein in a protein band^88^. As the SIFTER device is an open device design (vs. enclosed microchannels), protein is diffusively lost out of the microwell during cell lysis and out of the fractionation gel during electrophoresis. Such losses typically require proteins to be expressed at median copy number levels for mammalian proteins to be detectable in single-cell western blotting. While diffusive losses during SIFTER electrophoresis will be lower than in single-cell western blotting owing to efficient heat dissipation, protein fractionation inherently splits the amount of protein to be detected into the monomer and protein complex fractions. Thus, SIFTER likely requires proteins to be expressed above-median copy numbers for detection.

One major advantage of SIFTER over existing assays for protein complexes^89^, such as FRET or proximity labeling, is that SIFTER measures endogenous proteins without requiring cell modifications. Thus, we anticipate SIFTER will be valuable in the measurement of protein complexes from clinical specimens. For example, our group previously introduced isolated circulating tumor cells into a microwell array single-cell western blot device for protein profiling^37^. Circulating tumor cells are known to metastasize. With SIFTER, it would be informative to identify differentially expressed cytoskeletal protein complexes from circulating tumor cells to understand which protein complexes could be targets for small molecular inhibitors towards the prevention of metastasis.

For time-sensitive cytoskeletal re-organization or mechano-sensitive protein complexes within the cytoskeleton (e.g., stress fibers and focal adhesions), the fractionation gel functionality can be extended to also serve as a cell culture extracellular matrix. On-chip culture can assay adherent cells without the perturbation of trypsinization^90^. We anticipate that SIFTER can aid in evaluating snapshots of dynamic processes while cells are still adherent, such as cytoskeletal recovery from acute stress (e.g., heat shock, and hypoxia). In the present study, we trypsinized and gravity-settled heat-shocked cells for 10 min after the heat shock stress. The amount of time for cytoskeletal recovery from heat shock depends on the duration of the heat shock and cell type, as mouse fibroblasts only partially restore F-actin within 24 h after 1 h at 43 °C^82^. For shorter heat shock, or other stresses with faster recovery, growing and then stressing the cells on the SIFTER device will allow us to probe cytoskeletal protein-complex changes immediately after the stress, or at set times during the recovery. For mechano-sensitive cytoskeletal proteins, SIFTER may evaluate single-cell regulation of F-actin, MT, and IF in metastatic cancer cell subpopulations by quantifying dozens of cytoskeletal binding proteins with increased multiplexing by stripping and re-probing^88^. Looking ahead, SIFTER could assist drug screens targeting diverse protein interactions, and fundamental study of cellular stress responses underpinning invasive and heterogeneous cancer cells.

## Methods

### Chemicals

Tetramethylethylenediamine (TEMED, T9281), 40%T, 3.4%C acrylamide/bis-acrylamide (29:1) (A7802), N,N,N′,N′-, ammonium persulfate (APS, A3678), sodium deoxycholate (NaDOC, D6750), sodium dodecyl sulfate (SDS, L3771), bovine serum albumin (BSA, A7030), dithioerythritol (DTE, D8255), triton X-100 (X100), urea (U5378), β-mercaptoethanol (M3148), anhydrous magnesium chloride (MgCl_2,_ 814733) and dimethylsulfoxide (DMSO, D2438) were acquired from Sigma Aldrich. An Ultrapure Millipore filtration system provided deionized water (18.2 MΩ). PharmAgra Laboratories custom-synthesized N-[3-[(3-Benzoylphenyl)- formamido] propyl] methacrylamide (BPMAC). Phosphate buffered saline was purchased from VWR (10× PBS, 45001−130). Tris-glycine (10×) buffer was obtained from Bio-Rad (25 mM Tris, pH 8.3; 192 mM glycine, #1610734). Petroleum jelly (Cumberland Swan Petroleum Jelly, cat. no. 18−999−1829). Tris-HCl was obtained from Fisher Scientific (1 M, pH = 7.5; Corning MT46030CM), while 0.5 M Tris-HCl, pH 6.8 was purchased from Teknova (T1568). Photoinitiator 2,2-Azobis(2-methyl-N-(2-hydroxyethyl) propionamide) (VA-086) was acquired from FujiFilm Wako Pure Chemical Corporation. Gel Slick was purchased from Lonza (#50640). Tris Buffered Saline with Tween 20 (TBST-10×) was procured from Cell Signaling Technology (9997S). Paraformaldehyde (4% EM grade) was purchased from Electron Microscopy Sciences (157-4).

### Cell culture

All cell lines were authenticated by short tandem repeat profiling by the UC Berkeley Cell Culture facility and tested negative for mycoplasma. Naive U2OS cells were purchased from the UC Berkeley Cell Culture Facility. BJ fibroblasts expressing hTERT and Cas9 were provided by the Dillin lab. U2OS RFP-Lifeact cells were previously generated by the Kumar lab^91^ at UC Berkeley, and kindly provided for this study. MDA-MB-231 GFP-actin cells were kindly provided by the Drubin lab at UC Berkeley. BJ fibroblasts and U2OS (RFP-Lifeact and naive) cells were maintained in DMEM (11965, ThermoFisher Scientific) supplemented with 10% FBS (100-106, GeminiBio), 1% penicillin/streptomycin (15140-122, ThermoFisher Scientific), and 1% non-essential amino acids (11140-050, ThermoFisher Scientific), while MDA-MB-231 GFP-actin cells were maintained in the same media minus the 1% non-essential amino acids. All cells were cultivated in a humidified incubator in 5% CO_2_ kept at 37 °C. Cells were sub-cultured at ~80% confluency and detached with 0.05% Trypsin-EDTA (Gibco #25300-054) for 3 min. Each SIFTER assay was performed on a distinct single-cell suspension.

### Generation of RFP-Lenti MDA-MB-231 GFP-Actin cells

MDM-MB-231 GFP-actin cells were a kind gift from the laboratory of Dr. David Drubin. Genome editing was performed at the genomic locus by integrating TagGFP (see Source Data for sequence) at the genomic locus for *ACTB*. Verification of genome editing was performed via standard PCR and sequencing. Briefly, DNA was collected from cells using the Qiagen DNeasy Blood and Tissue Kit (69506) as per the manufacturer’s guidelines. 100 ng of genomic DNA was used for PCR and sequencing was performed using standard sanger sequencing (primers provided in Supplementary Table S2). A schematic for genome editing is provided in Supplementary Fig. S10. MDA-MB-231 GFP-actin cells were infected with lentivirus containing CD510B-1_pCDH-CMV-MCS-ED1-Puro (SystemBio) modified to carry TagRFP (see Source Data for sequence) under the CMV promoter.

### SIFTER assay (step-by-step protocol provided on Protocol Exchange)

Buffers and gel lid incubation: F-actin stabilization lysis buffer used was 10 mM Tris-HCl, 1% Triton X-100, 2 mM MgCl_2_, and 0.5 mM DTE (titrated to pH = 7.4)^92^. The DTE was added at the time of a given experiment. The depolymerization buffer was prepared as a 1.56× RIPA buffer such that upon addition of 8 M urea, the final buffer composition was 0.5× Tris-glycine, 0.5% SDS, 0.25% sodium deoxycholate, 0.1% Triton X-100, 8 M urea, pH = 8.3. Urea was added fresh at the time of the experiment and allowed to dissolve at 75 °C. Hydrogel lids (15%T, 3.3% C) were photopolymerized as previously described between Gel Slick-coated glass plates offset with a 500 μm spacer^26^. Hydrogel lids were incubated overnight at 4 °C in either the F-actin stabilization or the depolymerization buffer (before urea or DTE addition). Upon complete preparation of the urea-containing depolymerization buffer, the buffer was introduced to the gel lids in a water bath set to 75 °C and incubated for ~30 min before beginning the experiments. F-actin stabilization buffers and gel lids were kept at room temperature. Gel lids and buffers were only stored for up to 2 weeks, and buffer solution was never re-used.

Polyacrylamide fractionation gels (8%T and 3.3%C with 3 mM BPMAC incorporated) were polymerized on SU-8 micro-post molds as described elsewhere^27^. Trypsinization was performed for 3 min at 37 °C, and cells in PBS (10010049, Thermo Fisher Scientific, pH = 7.4, magnesium and calcium-free) settled in the microwell array for 10 min. Trypsinized cells were introduced to the microwell array in 1× PBS solution for passive gravity settling settling^27^. Every few minutes, the fractionation gel is gently slid back and forth to distribute cells across the gel. After 10 min, the gel is placed at a slight incline and excess cells are lightly rinsed off the gel surface with PBS. Each replicate experiment was run with a different 1 cm petri dish of freshly trypsinized cells in suspension.

For the fractionation separation, the fractionation gel device was pre-incubated in 10 mM Tris-HCl (pH = 7.5) briefly before the glass slide was adhered to the surface of a custom 3D-printed PAGE chamber with petroleum jelly. A custom heater with a 12 V PTC ceramic heating element (ELE147, Bolsen Tech) and PID temperature controller (ITC-106VH, Inkbird) was interfaced to the bottom surface of the PAGE chamber at 37 °C. The F-actin stabilization hydrogel lid was then applied to the array and cell lysis proceeded for 45 s before the electric field was applied (30 V cm^−1^, 45 s for 42 kDa actin in U2OS or BJ fibroblasts, or 60 s for 69 kDa GFP-actin from the GFP-actin cells; Bio-Rad Powerpac basic power supply). Proteins were blotted, or bound to the fractionation gel, by UV-induced covalent immobilization to the BPMAC incorporated in the fractionation gel (Lightningcure LC5, Hamamatsu, 100% power, 45 s). The electrode terminals were reversed, and the hydrogel lid was exchanged with depolymerization buffer gel hydrogel lid for 45 s. PAGE was performed for the same duration in the opposite direction before a final UV photo-immobilization step (same UV power and duration). The glass slide was peeled from the PAGE chamber, and the fractionation gel was washed in 1× TBST for at least 30 min to overnight prior to immunoprobing.

Immunoprobing was performed as previously described^27^, utilizing a rabbit anti-GFP antibody for GFP-actin (Abcam Ab290), rabbit anti-actin monoclonal antibody (Ab 200658 for BJ fibroblasts in Fig. 3), rabbit anti-actin monoclonal antibody (Abcam Ab 218787 for U2OS cells), mouse anti-vimentin monoclonal antibody (Abcam Ab8978) and rabbit anti-β-tubulin monoclonal antibody (Abcam Ab6046). See additional antibodies tested in Supplementary Table 1. Gels were incubated with 50 μl of 1:10 dilution of the stock primary antibody in TBST for 2 h and then washed 2× for 30 min in 1× TBST. Donkey Anti-Rabbit IgG (H+L) Cross-Adsorbed Secondary Antibody, Alexa Fluor 647-labeled (A31573, Thermo Fisher Scientific), Donkey Anti-Mouse IgG (H+L) Cross-Adsorbed Secondary Antibody, Alexa Fluor 555-labeled (A31570, Thermo Fisher Scientific), and Donkey Anti-Mouse IgG (H+L) Cross-Adsorbed Secondary Antibody, Alexa Fluor 647-labeled (A31571, Thermo Fisher Scientific) were used at a 1:20 dilution in TBST for a one-hour incubation after 5 min of centrifugation at 10,000 × *g*. Two more 30 min TBST washes were performed prior to drying the gels in a nitrogen stream and imaging with a laser microarray scanner (Genepix 4300A, Genepix Pro 7 software, Molecular Devices). When immunoprobing with rhodamine-labeled anti-actin Fab (see Supplementary Table 1) and Ab 200658, 1:5 dilutions were used. For the Fab, immunoprobing completed after the 2 h incubation and two 30 min wash in TBST. For multiplexed analysis of actin, vimentin, and β-tubulin protein complexes, actin and vimentin were immunoprobed together, the gels were chemically stripped^27^ and then re-probed for β-tubulin. Chemical stripping was performed for at least one hour at 55 °C. Gels were briefly rinsed in fresh 1× TBST three times and then washed in 1× TBST for at least one hour prior to re-probing.

Images were analyzed as described elsewhere^27^. Briefly, the images were median filtered utilizing the Remove Outliers macro in Fiji (pixel radius = 2 and threshold = 50 AFU; except for the data presented in Figs. 3b, c and  5). The images were then segmented, intensity profiles were generated for each separation lane and peaks were fit to a Gaussian curve. For fits with an *R*^2^ > 0.7 user-based quality control is performed, and the area under the curve is calculated within two peak widths from the center on the background-subtracted profile. The SNR of each peak is calculated with the signal taken as the amplitude of the Gaussian fit. Noise is calculated as the standard deviation of pixel intensities in a background region at the edge of the region of interest (aligned with two peak widths from the peak center location of the Gaussian). We report the area-under-the-curve for peaks with SNR > 3, a criterion used when determining a lower limit of detection for a semi-quantitative assay. Image analysis was performed in MATLAB R2019b.

### Temperature measurement in SIFTER

Temperature sensors (liquid crystal thermometers; Type C 30−60 °C with 5 °C intervals from ThermometerSite) were placed directly under the hydrogel lid (immersed in F-actin stabilization lysis buffer). The temperature was monitored while applying 30 V cm^−1^ across the electrodes of the electrophoresis chamber without interfacing with the custom heater.

Fluorescence imaging of cells in microwells, lysis, and PAGE: Imaging was performed via time-lapse epi-fluorescence microscopy on an Olympus IX50 and IX51 inverted epifluorescence microscope (and thus the custom heater was not used as it would block the illumination path through the PAGE chamber). The microscope was controlled using Metamorph software (Molecular Devices) and images were recorded with a CCD camera (Photometrics Coolsnap HQ2). The imaging setup included a motorized stage (ASI), a mercury arc lamp (X-cite, Lumen Dynamics), and a XF100-3 filter (Omega Optical) and 41017 (Chroma) for GFP and an XF111-2 filter for RFP (Omega Optical). Imaging was performed with a 10× magnification objective (Olympus UPlanFLN, NA 0.45 or UPLFLN10X2, NA 0.3) and 900 ms exposures with 1 s intervals with U2OS RFP-Lifeact, and 2 s exposure with 2 s intervals with MDA-MB-231 GFP-actin (1× pixel binning). Exposure times were lowered for lysis imaging to 600 ms.

### F-actin cell staining and drug treatment

Latrunculin A (Cayman Chemicals 10010630) was dissolved in DMSO as a 2 mM stock solution and stored at −20 °C until use. Jasplakinolide (Millipore-Sigma, 420107) was reconstituted in DMSO and stored at −20 °C for up to 3 months. Cells were incubated in the LatA for 60 min at 2 μM and in the Jpk for 120 min at 0.1 or 0.2 μM. Dosing concentration and duration to induce actin depolymerization were based on reported conditions for other cell types^62,93,94^ and verified qualitatively by phalloidin staining and fluorescence microscopy of adherent cells. The DMSO control cells were exposed to 0.1% DMSO in cell culture media for the same time as the drug-treated cells. Cells were fixed with 3.7% paraformaldehyde in 1× PBS (10 min at room temperature), and permeabilized with 0.1% Triton X-100 (for 5 min at room temperature and stained with Alexa Fluor 647-labeled phalloidin (20 min at room temperature, ThermoFisher Scientific, A22287).

Cells were imaged by epi-fluorescence with an Olympus IX70 inverted microscope controlled with Metamorph (Molecular Dynamics). Images were captured with an Andor iXon+ EMCCD camera (DU-885K-C00-#VP) with an Olympus LCPlanFl 40X (0.6 NA) objective, a mercury arc lamp (X-cite exacte, Lumen Dynamics), and a Chroma 49009 ET filter. Exposure time was 800 ms and pixel binning was 1×.

### Flow cytometry analysis of phalloidin-stained cells

Fixed cells were incubated in permeabilization buffer (0.1% Triton X-100 in PBS) at room temperature for 10 min. Cells were then spun down and incubated in staining solution (66 nM AlexaFluor 594 phalloidin in PBS supplemented with 2% BSA) at 4 °C for 30 min. Finally, cells were washed twice with PBS and analyzed with flow cytometry using BD LSRFortessa (and Flowjo v10.6 software). To analyze stained cells, single cells were gated by forward and side scatter (Supplementary Fig. S11). Only single cells were included in the fluorescence analysis.

### Heat shock treatment of cells

MDA-MB-231 GFP-actin RFP-lenti cells were incubated at 45 °C (VWR mini incubator, 10055-006) for heat shock, or at 37 °C in a humidified 5% CO_2_ incubator for controls for 1 h prior to trypsinization and gravity settling in the fractionation gel.

### Statistical analysis

Mann−Whitney test (with U test statistic) and Kruskal−Wallis test with post hoc Dunn’s test (Chi-squared test statistic), Spearman rank correlations, and QQ-plot generation with normal and gamma distributions were performed using pre-existing functions in MATLAB 2019b. All tests were two-sided. All boxplots include a centerline for the median, boxes at the 25th and 75th percentile, and whiskers that extend to the extremes of the data. Violin plots were generated in RStudio (Version 0.99.903) using the library Vioplot. The boxplot within the kernel density plot displays boxes at the 25th and 75th percentile, a point at the median, and whiskers that extend to the extremes of the data. Beeswarm plots used the library swarm.

### Cell fishing clustering analysis

Standardization is by row for both the LatA treated and DMSO control data sets (expression level, or Gaussian protein peak AUC, for each protein complex) with the mean at 0 and standard deviation of 1. Initial agglomerative hierarchical clustering was performed separately for the LatA treated and DMSO control data sets utilizing Euclidean distances, and the Ward linkage criterion (R version 3.6.1, NMF package). Distinct sub-clusters in the LatA treated data were further inspected as bait groups of cells inspired by the GeneFishing method described elsewhere^72^. We conducted an analogous analysis to GeneFishing, which we call Cell Fishing. Candidate cells from the DMSO control data sets were randomly split into subsamples of 100 cells, and each subsample was pooled together with the bait cells to form a sub-dataset. Semi-supervised clustering is applied to each sub-dataset using spectral analysis and a clustering algorithm based on the EM-fitted mixture Gaussian of two components model^95^ (R version 3.6.1, mclust package). The subsampling protocol was repeated 3000 times for a given bait set, and cells were considered fished out if they had a capture frequency rate of 0.999 or higher.

### Reporting summary

Further information on research design is available in the Nature Research Reporting Summary linked to this article.

## Supplementary information

Supplementary Information

Reporting Summary

## Data Availability

The data generated for this study are available in the Figshare repository with 10.6084/m9.figshare.c.5115779.v5. Source data for Figs. 1d−f, 2a−d,  3b−f,  4b−c, and  5b−d, and Figs. S1,  S3−8, and  S11 are provided with the paper. Source data are provided with this paper.

## Source data

Source Data

## Code availability

MATLAB functions to analyze protein expression levels from SIFTER gel images are available on Github (https://github.com/herrlabucb/SIFTER; 10.5281/zenodo.5091209)^96^ along with the CellFishing algorithm for R.
