# Supplementary material for: Measuring expression heterogeneity of single-cell cytoskeletal protein complexes
Source: Nat Commun. 2021 Aug 17;12:4969. doi: 10.1038/s41467-021-25212-3 (PMC8371148; doi:10.1038/s41467-021-25212-3)
Supplement: Supplementary file 1 — Supplementary Information [file 41467_2021_25212_MOESM1_ESM.pdf]

Measuring expression heterogeneity of single-cell cytoskeletal protein complexes

Julea Vlassakis, Ph.D.[1,†], Louise L. Hansen[1,†], Ryo Higuchi-Sanabria, Ph.D.[2], Yun Zhou[3], C. Kimberly Tsui, Ph.D.[2], Andrew Dillin, Ph.D.[2,4], Haiyan Huang, Ph.D.[5,6] and Amy E. Herr, Ph.D.[1,*]

1. Department of Bioengineering, University of California Berkeley, Berkeley, California 94720, United States.
2. Department of Molecular and Cell Biology, University of California Berkeley, Berkeley, California 94720, United States.
3. Division of Biostatistics, University of California Berkeley, Berkeley, California 94720, United States.
4. Howard Hughes Medical Institute, University of California Berkeley, Berkeley, California 94720, United States.
5. Department of Statistics, University of California Berkeley, Berkeley, California 94720, United States.
6. Center for Computational Biology, University of California Berkeley, Berkeley, California 94720, United States.

[†]. These authors contributed equally: Julea Vlassakis, Ph.D., and Louise L. Hansen
*Corresponding Author: Amy E. Herr, aeh@berkeley.edu

Supplementary Information

Supplementary Figure S1: Scatter plot and linear fit of protein molecular mass cutoff as a function of gel %T.

Supplementary Figure S2: Schematic representation of SIFTER and slab setup annotated with geometric parameters used for temperature model.

Supplementary Figure S3: Violin plots of the normalized log fold-change distributions of F-actin levels measured with flow cytometry and SIFTER.

Supplementary Figure S4: Boxplot of replicate DMSO control and LatA-treated U2OS cell measurements of F-actin by SIFTER from main text Figure 3F.

Supplementary Figure S5: Violin plots of F-actin, microtubule and intermediate filament expression levels from single cells detected in main text Figure 4.

Supplementary Figure S6: Correlation matrix for actin filament, microtubule and intermediate filament expression levels from single cells detected in main text Figure 4.

Supplementary Figure S7: Boxplots of replicate SIFTER assays performed at a set time post-trypsinization.

Supplementary Figure S8: Quantile-quantile plots of total actin and F-actin ratio single-cell distributions.

Supplementary Figure S9: Annotated false color fluorescence micrograph of a SIFTER device immunoprobed with a gasket fixture to test multiple antibodies in distinct regions of the gel.

Supplementary Figure S10: Schematic of MDA-MB-231 genome edit with GFP.

Supplementary Figure S11: Area density plots depicting flow cytometry gating strategy.

Supplementary Table S1: Summary of immunoprobing results with various antibodies in SIFTER.

Supplementary Table S2: Primers used in the study.

Supplementary Note 1: SIFTER buffer formulation and applicability to other cytoskeletal protein complexes.

Supplementary Note 2: Statistical analysis of flow cytometry and SIFTER distributions of F-actin levels in LatA versus DMSO control groups.

Supplementary Note 3: SIFTER immunoreagent screening.

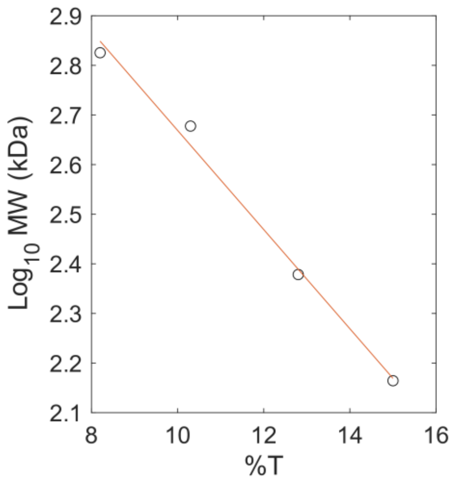

Supplementary Figure S1: Scatter plot of protein size excluded in blue native polyacrylamide gel electrophoresis (black circles) as a function of Total acrylamide concentration (%T, g/mL) as reported by Wittig et al.[1] The linear fit is shown as a red line with equation: y = -0.0999T + 3.6683 (r^2 = 0.992).

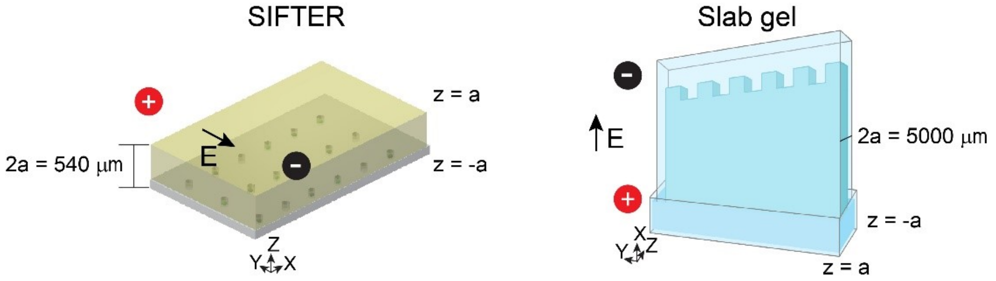

Supplementary Figure S2: Schematic representations of SIFTER and a slab gel setup with parameters used for estimates of temperature difference. Temperature difference between the edge of the conductor and different z positions is given by[2]: $\Delta T = E^2\sigma_c\left(\frac{a^2-z^2}{2k}\right)$ where E is the electric field (V m^{-1}), σ_c is the electrical conductivity (S m^{-1}), 2a is the cross-sectional thickness, and k is the thermal conductivity (W m^{-1} K^{-1}). We neglect an additional

term in the equation that accounts for heat transfer to a material incasing the conductor (thus we assume the width of the encasing material is zero). For E =3000 V m^{-1}, σ_c = 0.13 S m^{-1}, and approximating the conductor as water (given the high water volume fraction of polyacrylamide gel), k = 0.5918 we find ΔT=0.02 °K in SIFTER (at the fractionation gel or z = -0.00023), and ΔT = 6.18 °K in the slab gel (at the location of the sample in the slab gel, or z = 0). Slab gel schematic created with BioRender.com.

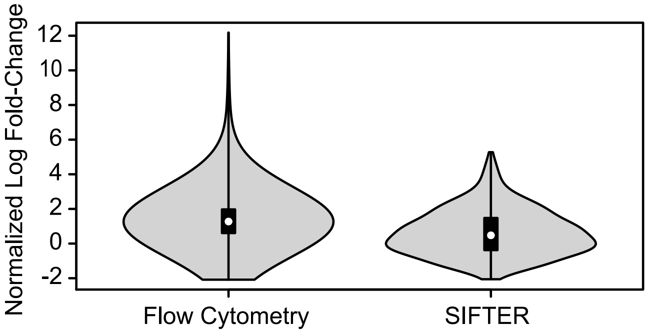

Supplementary Figure S3: Violin plot of the normalized log fold-change distributions in F-actin levels from flow cytometry (of trypsinized, fixed and phalloidin-stained U2OS cells; n = 9203 DMSO control cells and n = 5114 Latrunculin A (LatA)-treated cells from one experiment) and SIFTER (n = 911 DMSO control cells and n = 444 LatA-treated cells from N = 4 SIFTER devices) from main text Figure 3. Boxplot box edges are at 25th and 75th percentile, middle point is the median, and whiskers extend to minimum and maximum values of the data set. Normalization of DMSO control data to LatA data and subsampling results in a distribution with 44000 data points for flow cytometry and 444 data points for SIFTER, as described in Supplementary Note 2. Mann-Whitney p-value < 0.0001 and the 99% confidence interval for a shift in locations is [0.6306, 0.9304].

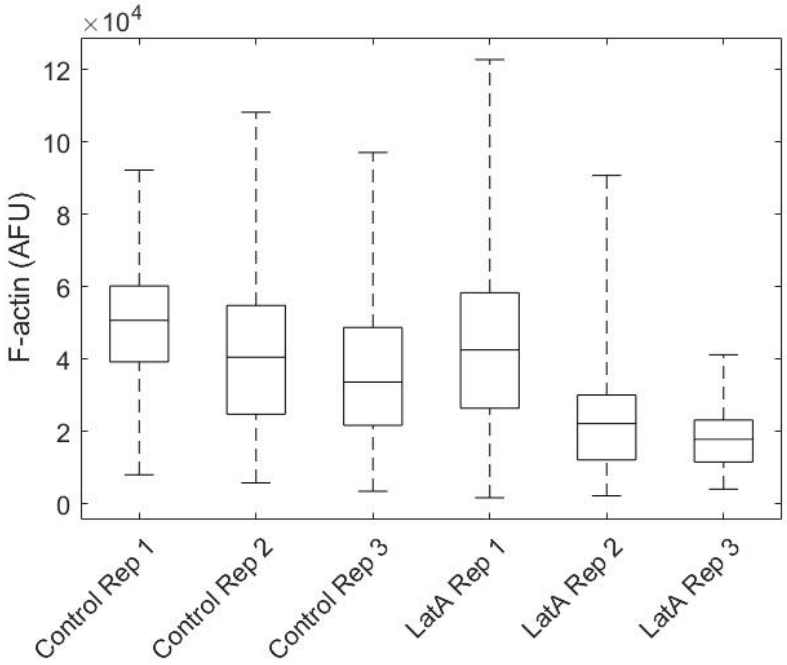

Supplementary Figure S4: Boxplot of F-actin quantified from DMSO Control and Latrunculin A (LatA) replicates (Rep) comprising main text Figure 3F. Kruskal-Wallis p-value < 0.005 for Control Rep 1-3 (n = 286, n = 287 and n = 338, respectively), and for LatA Rep 1-3 (n = 237, n = 97 and n = 110, respectively). Medians for Control Rep 1-3 are: 50749, 40601, and 33728, respectively. The DMSO Control mean median is 41693, and mean median coefficient of variation (CV) = 21%. Medians for LatA Rep 1-3 are: 42581, 22255 and 17885, respectively. The

LatA mean median is 27573 and mean median CV = 48%. Boxplot box edges are at 25th and 75th percentile, middle line is the median, and whiskers extend to minimum and maximum values of the data set.

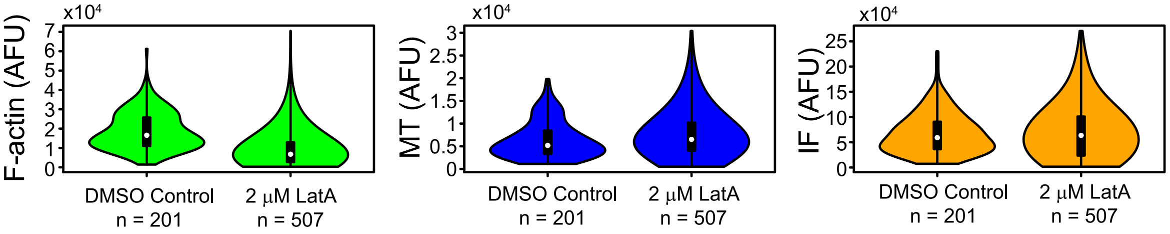

Supplementary Figure S5: Violin plots of F-actin (green), microtubule (MT, blue) and intermediate filament (IF, orange) expression levels with DMSO control or 2 μM LatA treatment. Boxplot box edges are at 25th and 75th percentile, middle point is the median, and whiskers extend to minimum and maximum values of the data set. Mann-Whitney (two-sided test) p-value is significant (*) p-value < 0.0001 for F-actin (DMSO control median = 16573 and LatA median = 6765). Mann-Whitney p-value = 0.0027 for MT (DMSO control median = 5187 and LatA median = 6517). The Mann-Whitney p=0.8917 for IF (DMSO control median = 59137 and LatA median = 63607).

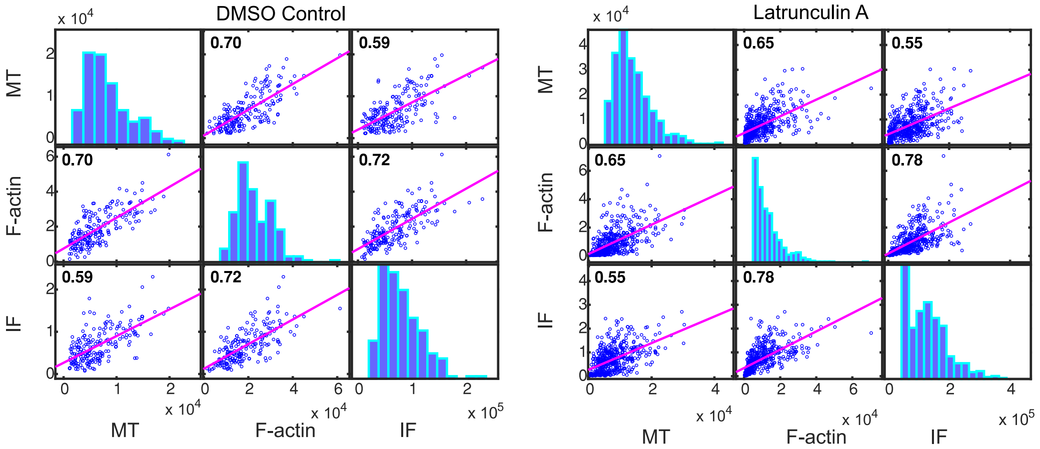

Supplementary Figure S6: Correlation matrices for DMSO control (n = 201 cells) and Latrunculin A treated cells (n = 507 cells) with histograms (blue bars) and scatter plots (blue circles). Protein complexes detected are microtubules (MT), F-actin and intermediate filaments (IF). Spearman ρ are shown in the upper left-hand corner of each scatter plot (p-value < 0.0001 for all correlations). Least-squares reference lines are shown in magenta with the slope equivalent to the correlation coefficient.

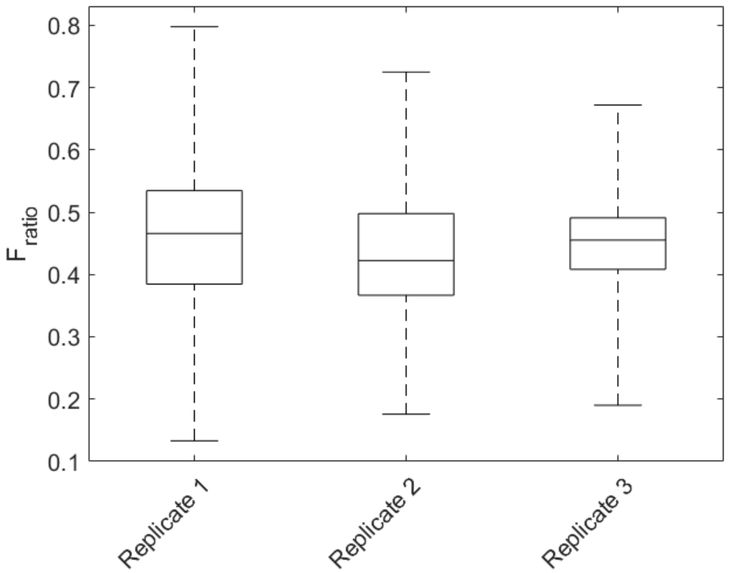

Supplementary Figure S7: Boxplots of F-actin ratio for three SIFTER replicates performed on MDA-MB-231 GFP-actin cells (three different batches of cells with constant 10-minute settling time post-trypsinization performed on the same day). Boxplot box edges are at 25th and 75th percentile, middle line is the median, and whiskers extend to minimum and maximum values of the data set. Replicate 1: n = 275; replicate 2: n = 193; replicate 3: n = 110. Kruskal-Wallis p-value = 0.0084; Dunn-Sidak post-hoc test for multiple comparisons p-values not significant except p = 0.006 for Replicate 1 (median = 0.47) vs. Replicate 2 (median = 0.42).

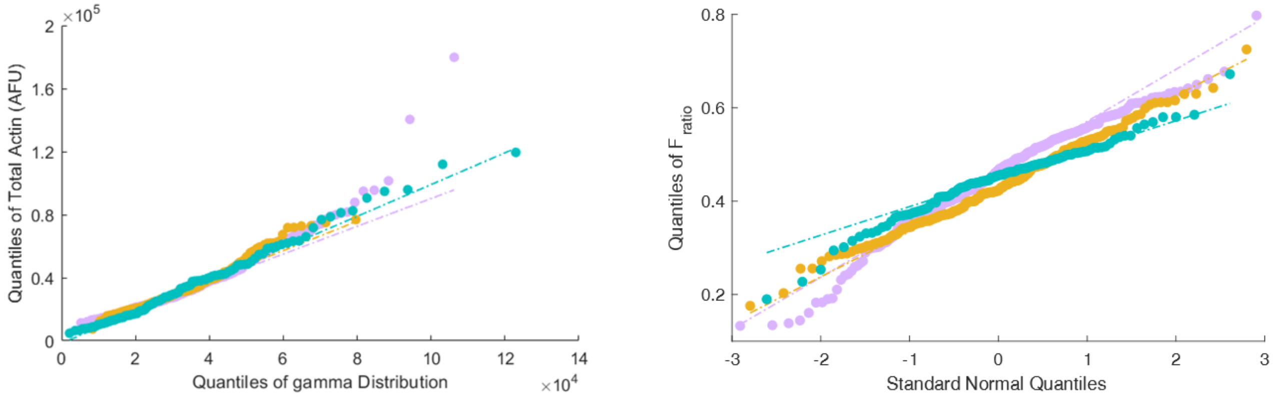

Supplementary Figure S8: Quantile-Quantile (QQ) plots from replicates SIFTER assays in Fig. S7. (a) Total actin (F+G) for each single cell versus a gamma distribution to each replicate (replicate 1: purple; replicate 2: orange; replicate 3: green). Single-cell total actin for each single cell is indicated with a circle symbol and dashed lines represent the fitted gamma probability density function ($f(x;\alpha,\beta)=\frac{x^{\alpha-1}e^{-\frac{x}{\beta}}}{\beta^{\alpha}(\alpha-1)!}$ for each replicate. Fit parameter α = 4.4, 6.9 and 2.2, and β = 8151, 4846, and 15361 for replicates 1, 2, and 3 respectively. (b) F-actin ratio (F_{ratio}) versus standard normal distribution.

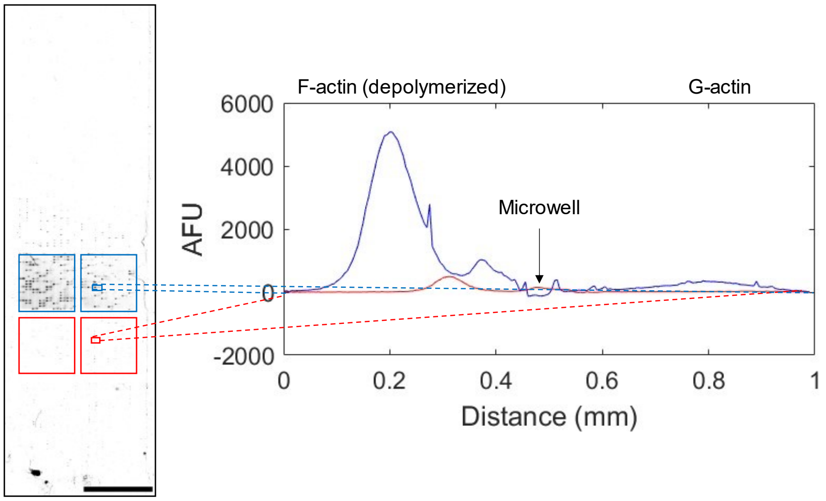

Supplementary Figure S9: Gasket-based antibody screening for full SIFTER fractionation gels. Left: false-color fluorescence micrograph of fractionation gels (BJ fibroblast F and G-actin separations) immunoprobed with different actin antibodies (Abcam Ab200658, blue; Abcam Ab198991, red). Scale bar is 10 mm.

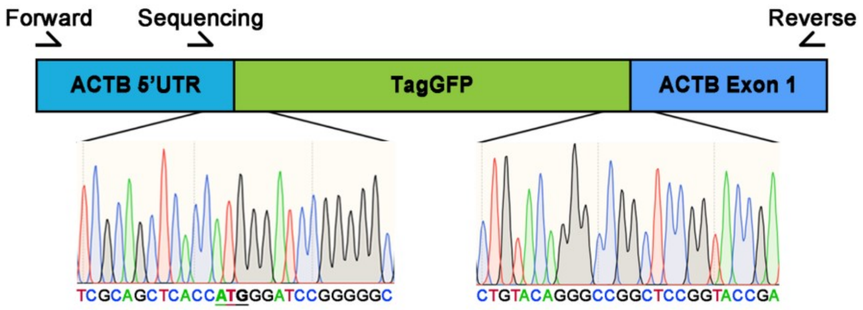

Supplementary Figure S10: Schematic of genome edit generating the GFP-actin fusion in the MDA-MB-231 cells. Actin gene (ACTB) exon, inserted GFP sequence (TagGFP) and untranslated region (UTR) shown.

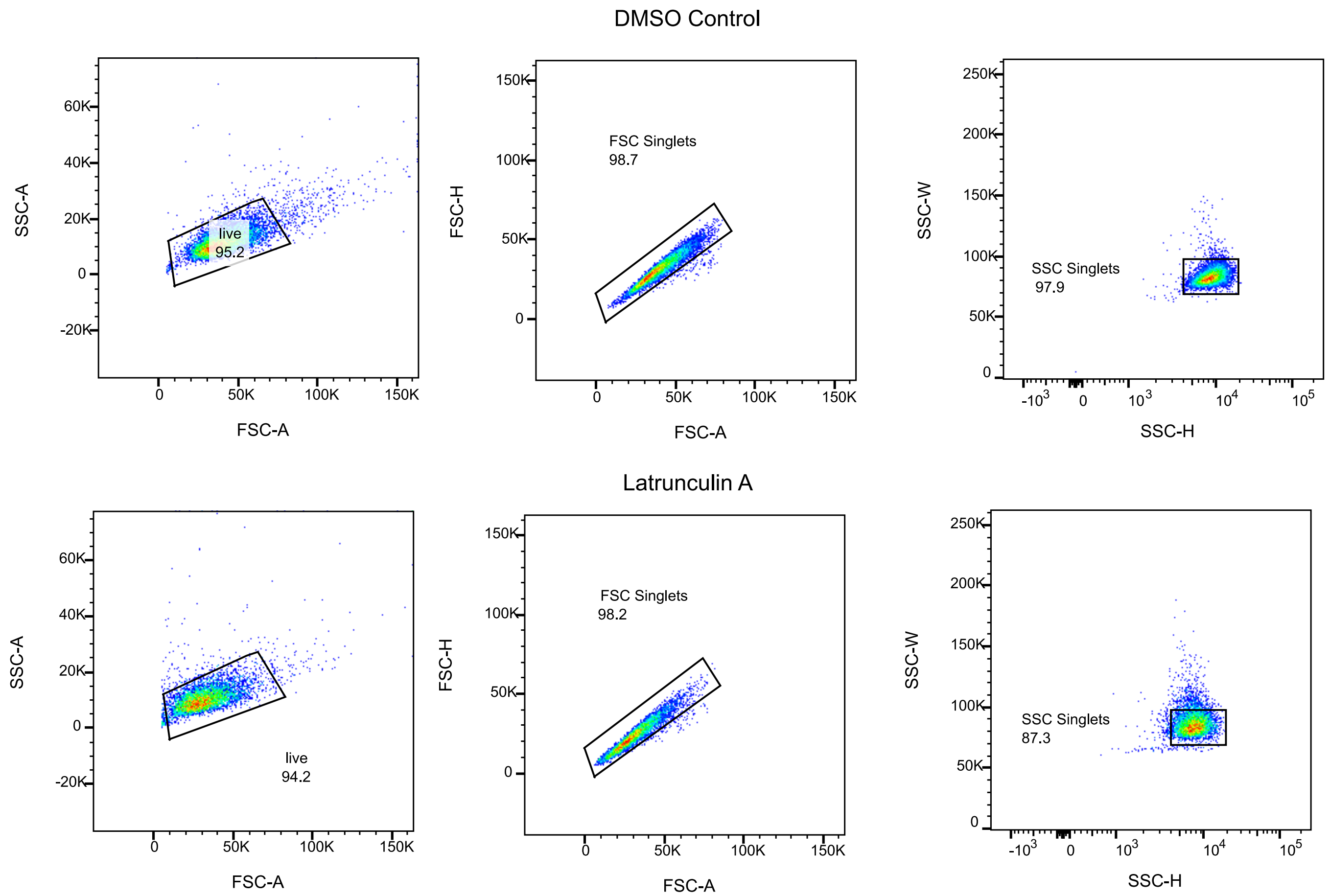

Supplementary Figure S11: Area density plots showing gating strategy for flow cytometry data presented in main text Figure 3d for the DMSO control and Latrunculin A-treated cells. Blue points correspond to the lowest densities and red are the highest densities of the color scale. Abbreviations: Side Scatter Area (SSC-A), Forward Scatter Area (FSC-A), Side Scatter Width (SSC-W), Side Scatter Height (SSC-H). Values provided on each plot are the percentage of events within each gate out of total events. Cells were first gated on FSC and SSC areas for live cells (i.e., live at the time of fixation), then gated on FSC (FSC singlets) and SSC (SSC singlets) for single cells. Fluorescence analysis was performed only on single cells.

Supplementary Table S1: Summary of immunoprobing results with various antibodies. Epitope information and valid applications indicated by the manufacturer are included. The 'Heater Status' column indicates whether the SIFTER assay was performed with the heater interfaced with the device during lysis and electrophoresis. Gels from the 'Without Heater' protocol were immunoprobed with 25 µl antibody solution instead of 50. Abbreviations: western blot (WB), enzyme-linked immunosorbent assay (ELISA), immunofluorescence (IF), immunohistochemistry (IHC), flow cytometry (flow), immunoprecipitation (IP), immunocytochemistry (ICC), amino acid (a.a.).

Vendor	Product #	Clonality	Epitope Info	Valid Applications	Separation results	Heater status
Millipore	MAB1501	Monoclonal (clone c4)	a.a. 50-70 (Chicken gizzard actin)	ELISA, IHC IF, ICC, & WB	F-actin band only (BJ fibroblasts)	With and without heater
CST	8456S	Monoclonal	C-terminus of β-actin (synthetic peptide)	WB, IF, IHC	F-actin band only (BJ fibroblasts)	Without heater
CST	8457	Monoclonal	Near N-terminus of β-actin (synthetic peptide)	WB, IF, Flow	F-actin band only (BJ fibroblasts)	With heater
CST	3700	Monoclonal	Near N-terminus of β-actin (synthetic peptide)	WB, IHC, IF, Flow	F-actin band only (BJ fibroblasts)	With heater
CST	4968S	Polyclonal	Residues around Asp244 (synthetic peptide)	WB, IHC	F-actin band only (BJ fibroblasts)	Without heater
Abcam	ab1801	Polyclonal	~residues 350 to C-terminus (peptide)	WB, IHC	No signal (BJ fibroblasts)	Without heater
Abcam	ab198991	Monoclonal	Synthetic peptide ~a.a. 300 to C-terminus	WB, IP	F-actin band only (BJ fibroblast)	With heater
Abcam	ab200658	Monoclonal	Synthetic peptide ~ a.a. 300 to C-terminus	WB, ICC, Flow	F-actin band with few G-actin bands (BJ fibroblast)	With heater
Abcam	ab218787	Monoclonal	Synthetic peptide corresponding to human actin	ICC, WB	F-actin band only (U2Os and BJ fibroblast)	With heater
Biorad	12004164	Unspecified; rhodamine-labeled Fab	Recombinant human beta β-actin expressed in e. Coli	WB	F-actin band and some G-actin bands (BJ fibroblasts)	Without heater

Supplementary Table S2: Primers used in the study.

Type of Primer	Sequence
Forward Primer for PCR	5’GGACTCAAGGCGCTAACTGC3’
Reverse Primer for PCR	5’ GGTACTTCAGGGTGAGGATGCC3’
Primer for Sanger Sequencing	5’GCTTCCTTTGTCCCCAATCTGG3’

Supplementary Note 1: We apply the F-actin stabilization buffer to the measurement of intermediate filament (IF) and microtubule (MT) cytoskeletal protein complexes. First, we note that MT and IF are relatively stable compared to F-actin. MT have depolymerization $t_{1/2}$ timescales of minutes[3] and IF experience subunit exchange ~10% over 7 hr[4]. Further due to the similarity in protein complex-stabilizing buffers for each cytoskeletal protein complex (Triton X-100 ~0.5-1.0%, pH ~6.7-7.4, and inclusion of 1 mM $MgCl_2$ for MT[5–7]), we determined the F-actin stabilization buffer employed in SIFTER could be usable for MT and IF fractionation.

Supplementary Note 2: For each of the two techniques, we converted the original data to the log scale, and normalized the LatA measurements by the mean and variance of the DMSO control. During normalization of the flow cytometry data set, sample size was accounted for by a repeated downsampling to match the sample size of SIFTER assay: we subsampled 444 points from 5114 flow cytometry LatA measurements and 911 points from 9203 flow cytometry DMSO control measurements. Next, we normalized the 444 subsampled LatA points, and repeated the subsampling and normalization protocol 100 times. The normalization procedure resulted in 444 SIFTER assay data points reflecting the log fold changes (DMSO control over LatA), and 44400 flow cytometry data points after pooling together the 100 subsamples. A Mann-Whitney test shows the normalized data in flow cytometry assay is significantly higher than for SIFTER assay, with a $p < 0.0001$ and 99% confidence interval of shift in locations being [0.6306, 0.9304]. This indicates that with a 99% high chance, the range [1.88, 2.54] will cover the ratio between the fold change in flow cytometry and the fold change in SIFTER measurement (DMSO control over LatA). Here 1.88=exp(0.6306), 2.54=exp(0.9304). The confidence interval suggests the fold change measured in flow cytometry assay is significantly higher.

Supplementary Note 3: We found that in cells expressing endogenous (not GFP-fused) actin, some immunoreagents detected depolymerized F- but not G-actin (Supplementary Table S1, Supplementary Figure S9). The performance of an antibody can vary for antigens under different assay conditions due to the changes in epitope accessibility. Antibodies recognize epitopes generally divided into two categories: linear epitopes and conformational epitopes[8,9]. In applications where the protein target is denatured during sample preparation, linear epitopes are preferred. In applications where protein targets are present in their native state, conformational epitopes are preferred. In SIFTER, the G-actin subpopulation is immobilized twice: once in native conditions, and once in denaturing conditions. The resultant conformation, secured by the BPMAC-immobilization, can be hypothesized to be a combination of the linear and conformational form. The orientation of the immobilized protein will affect antibody affinity, similar to protein on solid supports in protein microarrays[10,11]. Future work involving the mixing of antibodies against linear and conformational epitopes[12] could potentially recover more G-actin peaks.

Of note, a Fab fragment did yield G-actin immunoprobe signal in fibroblasts, while several full-length immunoreagents inconsistently detected G-actin in a subset of cells with F-actin signal. Lack of signal is

not likely due to detection sensitivity, as actin is present at millions of copies of protein per cell[13] (while the in-gel immunoprobing limit-of-detection is ~27,000 copies of protein[14]). We instead hypothesize sterics may influence epitope availability. The Fab fragment may bind to native G-actin immobilized to the gel, whereas full-length antibody probes are unable to do so (~3× larger molecular mass). Future investigations will aim to establish a protocol for in-gel monomeric protein denaturation prior to immobilization to ensure the epitope availability is consistent between monomeric and depolymerized protein complex fractions immobilized in the gel.

References:

1. Wittig, I., Beckhaus, T., Wumaier, Z., Karas, M. & Schägger, H. Mass estimation of native proteins by blue native electrophoresis: Principles and practical hints. *Mol. Cell. Proteomics* **9**, 2149–2161 (2010).
2. Cifuentes, A. & Poppe, H. Rectangular capillary electrophoresis: Some theoretical considerations. *Chromatographia* **39**, 391–404 (1994).
3. Caudron, N., Valiron, O., Usson, Y., Valiron, P. & Job, D. A reassessment of the factors affecting microtubule assembly and disassembly in vitro. *J. Mol. Biol.* **297**, 211–220 (2000).
4. Nöding, B., Herrmann, H. & Köster, S. Direct Observation of Subunit Exchange along Mature Vimentin Intermediate Filaments. *Biophys. J.* **107**, 2923–2931 (2014).
5. Heacock, C. S. & Bamburg, J. R. The quantitation of G- and F-actin in cultured cells. *Anal. Biochem.* **135**, 22–36 (1983).
6. Battaglia, R. A., Kabiraj, P., Willcockson, H. H., Lian, M. & Snider, N. T. Isolation of intermediate filament proteins from multiple mouse tissues to study aging-associated post-translational modifications. *J. Vis. Exp.* **2017**, 1–8 (2017).
7. Fourest-Lieuvin, A. Purification of tubulin from limited volumes of cultured cells. *Protein Expr. Purif.* **45**, 183–190 (2006).
8. Forsström, B. *et al.* Dissecting antibodies with regards to linear and conformational epitopes. *PLoS One* **10**, e0121673 (2015).
9. Zhao, Y. & Chait, B. T. Protein Epitope Mapping By Mass Spectrometry. *Anal. Chem.* **66**, 3723–3726 (1994).
10. Wang, J. *et al.* A versatile protein microarray platform enabling antibody profiling against denatured proteins. *Proteomics - Clin. Appl.* **7**, 378–383 (2013).
11. Liu, Y. & Yu, J. Oriented immobilization of proteins on solid supports for use in biosensors and biochips: a review. *Microchim. Acta* **183**, 1–19 (2016).
12. Einav, T. & Bloom, J. D. When two are better than one: Modeling the mechanisms of antibody mixtures. *PLoS Comput. Biol.* **16**, e1007830 (2020).
13. Li, J. J., Bickel, P. J. & Biggin, M. D. System wide analyses have underestimated protein abundances and the importance of transcription in mammals. *PeerJ* **2**, e270 (2014).
14. Kang, C.-C. *et al.* Single cell–resolution western blotting. *Nat. Protoc.* **11**, 1508–1530 (2016).
