# Supplementary material for: Measuring expression heterogeneity of single-cell cytoskeletal protein complexes
Source: Nat Commun. 2021 Aug 17;12:4969. doi: 10.1038/s41467-021-25212-3 (PMC8371148; doi:10.1038/s41467-021-25212-3)
Supplement: Supplementary file 2 — Reporting Summary [file 41467_2021_25212_MOESM2_ESM.pdf]

nature portfolio

Corresponding author(s): Amy E. Herr
Last updated by author(s): Jul 15, 2021

Reporting Summary

Nature Portfolio wishes to improve the reproducibility of the work that we publish. This form provides structure for consistency and transparency in reporting. For further information on Nature Portfolio policies, see our Editorial Policies and the Editorial Policy Checklist.

Statistics

For all statistical analyses, confirm that the following items are present in the figure legend, table legend, main text, or Methods section.

n/a	Confirmed	
☐	☒	The exact sample size (n) for each experimental group/condition, given as a discrete number and unit of measurement
☐	☒	A statement on whether measurements were taken from distinct samples or whether the same sample was measured repeatedly
☐	☒	The statistical test(s) used AND whether they are one- or two-sided<br>*Only common tests should be described solely by name; describe more complex techniques in the Methods section.*
☒	☐	A description of all covariates tested
☐	☒	A description of any assumptions or corrections, such as tests of normality and adjustment for multiple comparisons
☐	☒	A full description of the statistical parameters including central tendency (e.g. means) or other basic estimates (e.g. regression coefficient) AND variation (e.g. standard deviation) or associated estimates of uncertainty (e.g. confidence intervals)
☐	☒	For null hypothesis testing, the test statistic (e.g. F, t, r) with confidence intervals, effect sizes, degrees of freedom and P value noted<br>*Give P values as exact values whenever suitable.*
☒	☐	For Bayesian analysis, information on the choice of priors and Markov chain Monte Carlo settings
☐	☒	For hierarchical and complex designs, identification of the appropriate level for tests and full reporting of outcomes
☐	☒	Estimates of effect sizes (e.g. Cohen's d, Pearson's r), indicating how they were calculated

Our web collection on statistics for biologists contains articles on many of the points above.

Software and code

Policy information about availability of computer code

Data collection	Metamorph (v7.10.1.161 and v7.7.0.0, v7.8.0.0) and Genepix Pro (v7) software (Molecular Devices) was utilized for fluorescence image acquisition. Flowjo (v10.6) was used for flow cytometry data collection.
Data analysis	Analysis of SIFTER gels was performed using in-house scripts on MATLAB 2019b, and CellFishing analysis was carried out in R version 3.6.1 and the software for both analyses are available on GitHub (https://github.com/herrlabucb/SIFTER). Fiji (v1.51g) was used to false-color fluorescence micrographs and for SIFTER gel image pre-processing (e.g., alignment with Landmark Correspondences and median filtering with Remove Outliers). All other analysis was performed with built-in functions in MATLAB 2019b, RStudio version 0.99.903, and R version 3.6.1

For manuscripts utilizing custom algorithms or software that are central to the research but not yet described in published literature, software must be made available to editors and reviewers. We strongly encourage code deposition in a community repository (e.g. GitHub). See the Nature Portfolio guidelines for submitting code & software for further information.

Data

Policy information about availability of data

All manuscripts must include a data availability statement. This statement should provide the following information, where applicable:

- Accession codes, unique identifiers, or web links for publicly available datasets
- A description of any restrictions on data availability
- For clinical datasets or third party data, please ensure that the statement adheres to our policy

The data generated for this study are available in the Figshare repository with DOI: https://doi.org/10.6084/m9.figshare.c.5115779.v5. Source data for Fig. 1d-f, Fig. 2a-d, Fig. 3b-f, Fig. 4b-c, Fig. 5b-d, Fig. S1, Fig. S3-8 and Fig. S11 are provided with the paper.

Field-specific reporting

Please select the one below that is the best fit for your research. If you are not sure, read the appropriate sections before making your selection.

☒ Life sciences ☐ Behavioural & social sciences ☐ Ecological, evolutionary & environmental sciences

For a reference copy of the document with all sections, see nature.com/documents/nr-reporting-summary-flat.pdf

Life sciences study design

All studies must disclose on these points even when the disclosure is negative.

Sample size	Sample sizes in the SIFTER assay are determined by microwell occupancy (set by microwell diameter and cell seeding density) and the number of protein peaks that pass quality control on each device. While we did not perform power analysis prior to experiments owing to a lack of previously existing single-cell protein complex data for power analysis (e.g., with known standard deviations, etc.), we note that post-hoc sample size estimates confirm we assayed sufficient cell numbers to identify rare cell subpopulations. For example, to identify a 2% subpopulation with at least 1.5% precision and a 95% confidence interval would require at least 168 cells, and our cell subpopulation identification used 201-507 cells (e.g., in Figure 4).
Data exclusions	Some data are excluded during the quality control step of analysis of the SIFTER protein peaks as previously described (Kang. et al. Nat. Prot. 2016. 11, 1508-1530). Briefly, we discard images of protein peaks with visible damage to the gel or punctate autofluorescence in the region of the peak. Gaussian fits to the peaks must have an R-squared value greater than 0.7, as previously empirically determined, and the signal-to-noise ratio of each peak must be at least three.
Replication	The number of distinct SIFTER devices the assay was performed with is given with each data set (generally 2-4). In Figure 5c-d only one control SIFTER device is presented as replicates were originally collected with distinct batches of cells across different days, which we determined could not be pooled. However, the within-day F-actin ratio differences between control and heat shock conditions were consistent. At least two sets of images were collected per condition for fluorescence microscopy of phalloidin stained cells. Flow cytometry was performed once with n = 9203 control cells and n = 5114 LatA-treated cells. All attempts at replication were successful.
Randomization	Drug treatment and heat shock experiments were performed by either randomly assigning a number to each control and treatment group prior to beginning the experiment, or alternating control and treatment groups throughout the experiment (for SIFTER). For phalloidin staining of cells, the cells were deposited into wells of an 8-well culture slide or two and allowed to culture overnight. For drug treatment, one row of wells (or an entire slide) was randomly selected to be dosed with drug, while the other row (or the other entire slide) was dosed with DMSO vehicle control. For heat shock, one culture slide was randomly chosen to be put in the 45 °C incubator, while the other was maintained at 37 °C under regular cell culture conditions. No other experiments required allocation into experimental groups.
Blinding	Blinding was not performed and is unnecessary as the experimental protocol described in methods was performed consistently, and the quantitative analysis is not subjective. When the SIFTER assay is carried out, set times for cell trypsinization, cell settling, cell lysis, electrophoresis, UV photo-immobilizations and immunoprobing yield minimal risk for bias to impact the experimental result. During analysis, manual quality control (to identify peaks with punctate fluorescence spikes, etc.) is one potential source of bias, but R-squared and SNR thresholds assist with selection of Gaussian peaks with detectable signal.

Reporting for specific materials, systems and methods

We require information from authors about some types of materials, experimental systems and methods used in many studies. Here, indicate whether each material, system or method listed is relevant to your study. If you are not sure if a list item applies to your research, read the appropriate section before selecting a response.

Materials & experimental systems

n/a	Involved in the study
☐	☒ Antibodies
☐	☒ Eukaryotic cell lines
☒	☐ Palaeontology and archaeology
☒	☐ Animals and other organisms
☒	☐ Human research participants
☒	☐ Clinical data
☒	☐ Dual use research of concern

Methods

n/a	Involved in the study
☒	☐ ChIP-seq
☐	☒ Flow cytometry
☒	☐ MRI-based neuroimaging

Antibodies

Antibodies used	All antibodies used: rabbit anti-GFP antibody for GFP-actin (Abcam Ab290), mouse anti-actin monoclonal antibody (Millipore MAB1501), rabbit anti-actin monoclonal antibody (CST 8456S), rabbit anti-actin monoclonal antibody (CST 8457), mouse anti-actin monoclonal antibody (CST 3700), rabbit anti-actin polyclonal antibody (CST 4968S), rabbit anti-actin polyclonal antibody (Abcam Ab1801), rabbit anti-actin monoclonal antibody (Abcam Ab198991), rabbit anti-actin monoclonal antibody (Abcam Ab200658),

rhodamine-labeled anti-actin Fab (Biorad 12004164), rabbit anti-actin monoclonal antibody (Abcam Ab 218787), mouse anti-vimentin monoclonal antibody (Abcam Ab8978), rabbit anti-β-tubulin monoclonal antibody (Abcam Ab6046), Donkey Anti-Rabbit IgG (H+L) Cross-Adsorbed Secondary Antibody, Alexa Fluor 647-labeled (A31573, Thermo Fisher Scientific), Donkey Anti-Mouse IgG (H+L) Cross-Adsorbed Secondary Antibody, Alexa Fluor 555-labeled (A31570, Thermo Fisher Scientific) and Donkey Anti-Mouse IgG (H+L) Cross-Adsorbed Secondary Antibody, Alexa Fluor 647-labeled (A31571, Thermo Fisher Scientific

Validation

All primary antibodies from Abcam, Millipore and CST were validated for western blot according to the manufacturer's website (https://www.abcam.com/; https://www.cellsignal.com/browse/primary-antibodies; https://www.emdmillipore.com/US/en/product/Anti-Actin-Antibody-clone-C4,MM_NF-MAB1501#anchor_Applications;). The anti-actin Fab from Biorad is intended for use in western blotting as indicated on the manufacturer's website (https://www.bio-rad.com/en-us/category/fluorescent-western-blotting-antibodies?ID=OKNWV64VY).

Abcam general western blot validation statement (https://www.abcam.com/primary-antibodies/how-we-validate-our-antibodies#Western%20blot): “Antibodies are validated in western blot using lysates from cells or tissues that we have identified to express the protein of interest. Once we have determined the right lysates to use, western blots are run and the band size is checked for the expected molecular weight. We will always run several controls in the same western blot experiment, including positive lysate and negative lysate (if possible, Figure 2).

When possible, we also include knock-out (KO) cell lines as a true negative control for our western blots. We are always increasing the number of KO-validated antibodies we provide. In addition, we run old stock alongside our new stock. If we know the old stock works well, this also acts as a suitable positive control.

If the western blot result gives a clear clean band and we are happy with the result from the control lanes, these antibodies will be passed and added to the catalog.”

Additional details on Abcam primary antibodies used:
Abcam Ab 290 from the manufacturer (https://www.abcam.com/gfp-antibody-ab290.html?productWallTab=Abreviews&applications=69): "On Western blot the antibody detects the GFP fraction from cell extracts expressing recombinant GFP fusion proteins and has also been shown to be useful on mouse sections fixed with formalin.”
Abcam Ab 290 CiteAb profile: https://www.citeab.com/antibodies/575390-ab290-anti-gfp-antibody

Abcam Ab 1801 from the manufacturer (https://www.abcam.com/actin-antibody-loading-control-ab1801.html): “This antibody detects a single clean band in Human, Mouse, Rat, Chicken and Drosophila samples. In Xenopus laevis a secondary band is detected at about 30kDa. We are unsure whether this is cross-reaction with another actin isoform or merely non-specific. In Cow a doublet is detected, which probably represents different forms of actin.”
Abcam Ab 1801 CiteAb profile: https://www.citeab.com/antibodies/708757-ab1801-anti-actin-antibody-loading-control

Abcam Ab 198991 from the manufacturer (https://www.abcam.com/actin-antibody-epr16770-ab198991.html): “Detects a band of approximately 42 kDa (predicted molecular weight: 42 kDa).” Positive controls include “WB: HeLa, 293, C6, RAW 264.7, PC-12 and NIH/3T3 whole cell lysates...”
Abcam Ab 198991 CiteAb profile: https://www.citeab.com/antibodies/2434472-ab198991-anti-actin-antibody-epr16770?des=793dcbc14e323433

Abcam Ab 200658 from the manufacturer (https://www.abcam.com/actin-antibody-epr16875-ab200658.html): “Detects a band of approximately 42 kDa (predicted molecular weight: 42 kDa).” Positive controls include “WB: Jurkat, HepG2, HeLa, UMNSAH/DF-1, C6 and RAW 264.7 cell lysates...”
Abcam Ab 200658 CiteAb profile: https://www.citeab.com/antibodies/2927653-ab200658-anti-actin-antibody-epr16875?des=80d6eef8157fc2dc

Abcam Ab 218787 from the manufacturer (https://www.abcam.com/actin-antibody-igx3831r-3-ab218787.html): “Detects a band of approximately 42 kDa (predicted molecular weight: 42 kDa). Positive controls include “WB: Human and mouse colon, HeLa, Jurkat, NIH3T3, PANC1, C2C12 whole cell lysates and human skeletal muscle...”

Abcam Ab 8978 from the manufacturer (https://www.abcam.com/vimentin-antibody-rv202-cytoskeleton-marker-ab8978.html): “This antibody reacts exclusively with vimentin, which is expressed in mesenchymal cells and mesenchymal derived tumors e.g. lymphoma, sarcoma and melanoma.” Positive controls include “WB: NIH-3T3 and normal human dermal fibroblasts cell extracts.”
Abcam Ab 8978 CiteAb profile: https://www.citeab.com/antibodies/759383-ab8978-anti-vimentin-antibody-rv202-cytoskeleton?des=c26db556db84655e

Abcam Ab 6046 from the manufacturer (https://www.abcam.com/beta-tubulin-antibody-loading-control-ab6046.html): “This antibody detects a single clean band at 50kD representing beta Tubulin. This band is significantly reduced by using peptide blocking.” Positive controls include “WB: HeLa, A431, MCF7, and 293 cell lysates...”
Abcam Ab 6046 CiteAb profile.: https://www.citeab.com/antibodies/714353-ab6046-anti-beta-tubulin-antibody-loading-control?des=63bc5dfe46199346
Additionally our lab has previously use this antibody in single-cell western blotting (https://doi.org/10.1038/nmeth.2992).

CST general western blot validation statement (https://www.cellsignal.com/about-us/our-approach-process/antibody-validation-western-blotting): “CST™ antibodies are produced in-house and validated extensively according to a rigorous protocol.
Validation Steps Include
• Examination of several cell lines and/or tissues of known expression levels allows accurate determination of species cross-reactivity and verifies specificity.
• Treatment of cell lines with growth factors, chemical activators or inhibitors, which induce or inhibit target expression, verifies specificity. Phosphatase treatment confirms phospho-specificity.
• The use of siRNA transfection or knockout cell lines verifies target specificity.
• Side-by-side comparison of lots to ensures lot-to-lot consistency.
• Optimal dilutions and buffers are predetermined, positive and negative cell extracts are specified, and detailed protocols are

already optimized, saving valuable time and reagents."

Additional details on primary CST antibodies used:
CST 8456 from the manufacturer (https://www.cellsignal.com/products/primary-antibodies/pan-actin-d18c11-rabbit-mab/8456): "Pan-Actin (D18C11) Rabbit mAb recognizes endogenous levels of total actin protein (all isoforms). Species Reactivity: Human, Mouse, Rat, Monkey." Strong band between 40-50 kDa shown on image of western blot validation data from the manufacturer with cell lysates from A-431, 293, MCF7 and COS-7 cells. MCF7 cells also have faint bands below 30 kDa.
CST 8456 CiteAb profile: https://www.citeab.com/antibodies/125241-8456-pan-actin-d18c11-rabbit-mab?des=5bae6952ec71dd3a

CST 3700 from the manufacturer (https://www.cellsignal.com/products/primary-antibodies/b-actin-8h10d10-mouse-mab/3700): "β-Actin (8H10D10) Mouse mAb detects endogenous levels of total β-actin protein. Due to the high sequence identity between the cytoplasmic actin isoforms, β-actin and cytoplasmic γ-actin, this antibody may cross-react with cytoplasmic γ-actin. It does not cross-react with α-skeletal, α-cardiac, α-vascular smooth, or γ-enteric smooth muscle isoforms. Species Reactivity: Human, Mouse, Rat, Hamster, Monkey, Dog." Single strong band between 40-50 kDa shown on image of western blot validation data from the manufacturer with cell lysates from COS, HeLa, C2C12, C6 and CHO cells.
CST 3700 CiteAb profile: https://www.citeab.com/antibodies/123338-3700-actin-8h10d10-mouse-mab?des=e80d5580807148e0

CST 4968 from the manufacturer (https://www.cellsignal.com/products/primary-antibodies/pan-actin-antibody/4968): "Specificity / Sensitivity Pan-Actin Antibody detects endogenous levels of total actin (all isoforms). The antibody also detects the 30 kDa actin fragment cleaved at glutamate 107. Species Reactivity: Human, Mouse, Rat, Monkey, Zebrafish." Strong band between 40-50 kDa and faint band at 30 kDa shown on image of western blot validation data from the manufacturer with cell lysates from HeLa, L929, C6, COS.
CST 4968 CiteAb profile: https://www.citeab.com/antibodies/124952-4968-pan-actin-antibody?des=bd570f5354cb6d1b

In this work, antibodies were used in polyacrylamide gel electrophoresis separations which would allow for identification of additional off-target peaks of lower or higher molecular mass than the target.

Eukaryotic cell lines

Policy information about cell lines

Cell line source(s)	MDA-MB-231 GFP-actin cells, U2OS RFP-LifeAct cells and BJ fibroblast cells expressing hTERT and Cas9 were generously provided by the laboratories of Dr. David Drubin, Dr. Sanjay Kumar and Dr. Andrew Dillin, respectively (all at UC Berkeley). RFP-lenti MDA-MB-231 GFP-actin cells were generated as described in the Methods.
Authentication	All cell lines were authenticated by short tandem repeat profiling by the UC Berkeley Cell Culture Facility.
Mycoplasma contamination	All cell lines tested negative for mycoplasma infection by the UC Berkeley Cell Culture Facility.
Commonly misidentified lines (See ICLAC register)	No commonly misidentified cell lines were used in this study.

Flow Cytometry

Plots

Confirm that:

☐ The axis labels state the marker and fluorochrome used (e.g. CD4-FITC).

☒ The axis scales are clearly visible. Include numbers along axes only for bottom left plot of group (a 'group' is an analysis of identical markers).

☐ All plots are contour plots with outliers or pseudocolor plots.

☒ A numerical value for number of cells or percentage (with statistics) is provided.

Methodology

Sample preparation	U2OS cells were fixed in 3.7% paraformaldehyde in 1X PBS for 10 minutes and permeabilized in 0.1% Triton X-100 in 1X PBS for 10 minutes. Cells were spun down and stained in 66 nM AlexaFluor 594-labeled phalloidin with 2% BSA in 1X PBS for 30 minutes. Cells were washed twice in 1X PBS
Instrument	BD LSRFortessa
Software	Flowjo (v10.6)
Cell population abundance	All analyzed samples are pure samples that has undergone identical staining procedure.
Gating strategy	Cells were gated on FSS/SCC for live, single cells prior to fluorescence analysis.

☒ Tick this box to confirm that a figure exemplifying the gating strategy is provided in the Supplementary Information.
